# DCBLD1 Promotes Lung Tumorigenesis by Inhibiting PTP1B Dephosphorylation of EGFR

**DOI:** 10.7150/ijbs.112100

**Published:** 2026-01-01

**Authors:** Ying Liu, Yangyang Li, Xiaowei Quan, Jiayi Zhang, Zhicong Wang, Zhaoyuan Hou, Herbert Yu, Haipeng Liu, Tengteng Zhu, Biyun Qian

**Affiliations:** 1 Hongqiao International Institute of Medicine, Shanghai Tongren Hospital and School of Public Health, Shanghai Jiao Tong University School of Medicine, Shanghai 200025, China.; 2 Institute of Clinical Medicine, Shanghai Jiao Tong University School of Medicine, Shanghai 200025, China.; 3 Faculty of Basic Medicine, Shanghai Jiao Tong University School of Medicine, Shanghai 200025, China.; 4 Cancer Epidemiology Program, University of Hawaii Cancer Center, Honolulu 96813, HI, USA.; 5 Innovation and Incubation Center, Shanghai Pulmonary Hospital, Tongji University School of Medicine, Shanghai 200433, China.

**Keywords:** DCBLD1, EGFR, PTP1B, lung adenocarcinoma, tumorigenesis

## Abstract

Lung adenocarcinoma (LUAD) progression involves multistep molecular pathogenesis, with many critical mediators of malignant transformation yet to be fully characterized. Building upon our previous discovery of discoidin, CUB and LCCL domain containing 1 (DCBLD1) as a novel LUAD risk-associated gene, we systematically investigated its function and underlying mechanisms in LUAD. Intriguingly, DCBLD1 overexpression promotes cellular transformation in both bronchial epithelial cells and EGFR^L858R^ alveolar type II organoids, while its deficiency in DCBLD1^-/-^ mice significantly suppresses LUAD initiation. Mechanistic studies revealed that DCBLD1 drives oncogenesis through direct interaction with EGFR. Specifically, the intracellular domain of DCBLD1 competitively binds to EGFR, displacing the critical negative regulator PTP1B phosphatase. This displacement impairs EGFR dephosphorylation, leading to sustained receptor activation and subsequent hyperactivation of downstream PI3K/AKT and MAPK signaling cascades. The sustained signaling activation produces significant clinical implications for LUAD treatment. In therapeutic studies, DCBLD1 knockdown demonstrated substantial antitumor effects in both patient-derived organoid and xenograft models, independent of EGFR mutation status. These findings position DCBLD1 as a promising therapeutic target for LUAD patients, offering a potential strategy that complements current EGFR mutation-based approaches.

## Introduction

Lung cancer is an aggressive malignancy characterized by high morbidity and mortality rates. Non-small cell lung cancer (NSCLC) accounts for approximately 85% of all cases, with lung adenocarcinoma (LUAD) being the most prevalent subtype 1. Given the complex genetic landscape of this disease, researchers have been increasingly focused on identifying genetic variations that may influence cancer susceptibility and progression 2,3.

Genome-wide association studies (GWAS) have revealed over 50 lung cancer susceptibility loci 4, with our previous research identifying the discoidin, CUB and LCCL domain containing 1 (DCBLD1) promoter single nucleotide polymorphism (SNP) rs17079281 C > T variant associated with reduced LUAD risk (OR = 0.86; 95% CI: 0.80-0.92). Mechanistically, the T allele suppresses DCBLD1 expression by enhancing YY1 binding affinity, thereby conferring a protective effect against LUAD 5. The inherent irreversibility of genomic variations presents significant challenges in preventing their role in LUAD initiation 6. Consequently, a comprehensive understanding of DCBLD1's function and its underlying mechanisms in LUAD tumorigenesis becomes crucial.

Emerging evidence highlights DCBLD1's broader clinical significance, with genetic polymorphisms linked to LUAD, severe chronic obstructive pulmonary disease, and emphysema 7,8. Moreover, elevated DCBLD1 expression correlates with unfavorable prognoses across multiple cancer types, including NSCLC, breast cancer, and head and neck squamous cell carcinoma 9,10. These preliminary findings suggest DCBLD1 as a promising yet understudied therapeutic target, particularly in LUAD, where emerging molecular insights are increasingly critical to advancing precision oncology.

Personalized treatment strategies for LUAD are predominantly guided by the precise molecular characterization of genetic mutations 11,12. Specifically, for patients with EGFR mutant (Mut), EGFR tyrosine kinase inhibitors (TKIs) have emerged as the standard first-line intervention. While patients receiving EGFR TKIs treatment inevitably develop acquired resistance, this substantially compromises the long-term efficacy 13. For EGFR wild-type (WT) LUAD patients, the therapeutic landscape is primarily limited to immunotherapy and conventional chemotherapy 14,15. This clinical landscape underscores the urgent need for innovative targeted therapeutic approaches.

In this study, we identified DCBLD1 as a key oncogenic driver in LUAD by demonstrating its role in maintaining EGFR signaling through competitive inhibition of PTP1B-mediated dephosphorylation. Through extensive validation in cellular, organoid, and transgenic mouse models, the research established DCBLD1 as a promising therapeutic target that could benefit LUAD patients regardless of their EGFR mutation status.

## Materials and Methods

### Cell culture and treatments

Human LUAD cell lines (A549, H1299 and PC9), immortalized normal human bronchial epithelial cell line (BEAS-2B) and human embryonic kidney cell line (HEK-293T) were obtained from the Cell Bank of the Chinese Academy of Sciences (Shanghai, China). All cell lines were cultured in DMEM (BasalMedia, Shanghai, China) supplemented with 10% FBS (Cytiva, Uppsala, Sweden) and 1% penicillin-streptomycin medium (Yeasen, Shanghai, China) under humidified 5% CO_2_ at 37 °C. Cells were used between passages 5 and 15 for experiments. To stimulate or suppress EGFR activity, cells were treated with either 100 ng/mL EGF (Sino Biological lnc., Beijing, China) for 15 min or gefitinib (Selleck, Houston, TX, USA), respectively.

### Human samples

The LUAD specimens used for establishing patient-derived organoid (PDO) and xenograft (PDX) models were obtained from patients who underwent radical tumor resection at Shanghai Tongren Hospital. The patient selection for this study was based on ARMS-PCR confirmed EGFR WT or EGFR Mut (exon 19 deletion) status. None of the patients received local or systemic treatment. The study protocol and tissue collection procedures were approved by the Ethics Committee (Ethics Code 2021-015), and all participants provided written informed consent.

### Analysis of public and independent cohorts

DCBLD1 expression data were retrieved from TCGA (https://portal.gdc.cancer.gov/) and GEO (https://www.ncbi.nlm.nih.gov/geo/) databases. Differential expression analysis between tumor and adjacent normal tissues was performed using Student's t-test. For survival analysis, patients were stratified into high and low expression groups using the surv_cutpoint function (survminer package, R 4.3.2). To further validate findings from public databases, a secondary analysis was conducted using an independent LUAD cohort (n = 78) from a previously published study 5. Additionally, a total of 100 LUAD specimens, comprising 50 EGFR wild-type and 50 EGFR-mutant cases, were obtained from Shanghai Pulmonary Hospital with approval from the Institutional Ethics Committee (Ethics Code K25-588). These samples were subjected to immunohistochemistry (IHC) staining for DCBLD1 and pEGFR. Protein expression levels were semi-quantitatively assessed using a combined scoring system based on staining intensity and density.

### Genetically engineered mouse models

To obtain EGFR^L858R/+^ mice, SFTPC-IRES-iCre mice (Strain NO. T004715) and H11-CAG-LSL-EGFR-(L858R) mice (Strain NO. T007734) purchased from GemPharmatech (Nanjing, China) were crossed. DCBLD1 KO mice on a C57BL/6J background were engineered by Nanjing GemPharmatech Co., Ltd using CRISPR/Cas9 technology. Briefly, gRNA was designed to create a ~300bp deletion covering the coding region from exon 3 to exon 5. Genotyping was performed using specific primers (Table S1) to amplify the DNA, followed by agarose gel electrophoresis for detection.

### Isolation of mouse AT2 cells

Alveolar type II (AT2) cells were isolated from EGFR^L858R/+^ mice as previously described, with minor modifications 16. After anesthesia, dispase (Corning, NY, USA) was infused intratracheally, and the lung tissues were minced and incubated in a mixture of DNase I (Sigma-Aldrich, St. Louis, MO, USA) and collagenase/dispase (Roche, Basel, Switzerland) at 37 °C for 45 minutes. The resulting cell suspension was filtered and centrifuged, followed by red blood cell lysis. Cells were then resuspended in PBS with 10% FBS and stained for FACS sorting using CD45, CD31, and EpCAM (BD Pharmingen, San Diego, CA, USA) antibodies to isolate CD45^-^, CD31^-^, EpCAM^+^ cells.

### Acquisition of patient LUAD cells

Briefly, LUAD tissues from EGFR-WT or EGFR-mutant LUAD patients were minced and washed in cold DPBS (BasalMedia, Shanghai, China). One-third of the sectioned samples were incubated with tissue digestion buffer (Ombio, Shenzhen, China) at 37 °C with intermittent agitation, while the remaining tissue was reserved for PDX model establishment. Trypsinization was stopped by adding organoid protection buffer (Ombio, Shenzhen, China) upon observation of sufficient single cells or cell clusters.

### Organoid cultivation and passaging

For both mouse and human organoid cultures, cell suspensions were mixed with Matrigel (Corning, NY, USA) at a 1:1 volume ratio and seeded at 50 μL per well in 24-well plates. After Matrigel solidification, 500 μL specific culture media were added: Mouse Lung Organoid Culture Medium (Ombio, Shenzhen, China) for mouse AT2-derived organoids, and Human Lung Cancer Organoid Culture Medium (Ombio, Shenzhen, China) for patient-derived lung cancer organoid. Culture medium was refreshed every 3 days. For passaging, organoids were harvested by incubating the Matrigel-embedded cultures in cold DPBS at 4 °C for 30 minutes to dissolve the Matrigel. Following centrifugation and Matrigel removal, organoids were enzymatically dissociated using 500 μL TrypLE (Gibco, Grand Island, NY, USA) at 37 °C for 3 minutes, then mechanically dispersed into smaller clusters. Organoids were passaged at a 1:5 dilution every 2 weeks.

### Plasmid construction

For gene overexpression, full-length sequences of DCBLD1, DCBLD2, EGFR and PTP1B were PCR amplified from cDNA using custom-designed primers fused with the Flag or HA tag at the C-terminus. Subsequently, these sequences were cloned into the linearized vectors using ClonExpress Ultra One Step Cloning Kit (Vazyme, Nanjing, China). Truncated variants of DCBLD1 and EGFR were generated by reverse PCR using the Mut Express MultiS Fast Mutagenesis Kit V2 (Vazyme, Nanjing, China). For gene knockdown, shDCBLD1 and shPTP1B were generated with the custom-designed shRNA oligos and PLKO.1 backbone. All constructs were validated by Sanger sequencing (Sangon Biotech, Shanghai, China). Primer sequences are listed in Table S1.

### Lentiviral production and transduction

HEK-293T cells were co-transfected with target gene constructs (overexpression or knockdown) and lentiviral packaging plasmids (psPAX2 and pMD2.G) using PEI MW25000 (Polysciences, Warrington, PA, USA). Lentiviral supernatants were harvested at 48 and 72 hours post-transfection and filtered through 0.45 μm membranes. Cells were then transduced with the collected lentiviruses in the presence of polybrene, followed by puromycin selection.

Organoids were transduced following a previously described method 17. Briefly, matrigel was added to each well and allowed to solidify before overlaying the organoid-lentivirus mixture. After 16 hours, medium containing dead organoids was removed, and an additional layer of matrigel was added to cover the attached organoids. Once the matrigel solidified, organoid culture medium was added. Puromycin selection was initiated several days post-infection.

### Cell viability assay

Cell viability was evaluated using Cell Counting Kit-8 (ApexBio Technology, Boston, USA). Briefly, cells subjected to different treatments were seeded in 96-well plates at a density of 8×10³ cells per well. At the indicated time points, 10 μL of CCK-8 reagent was added per well. Absorbance was measured at 450 nm using a microplate reader (Thermo Scientific, Waltham, MA, USA).

### Colony formation assay

For bronchial epithelial cells, the bottom layer was prepared using 0.6% agar in DMEM supplemented with 10% FBS, then transferred into 6-well plates. The mixture was incubated at room temperature for 30 minutes to solidify. BEAS-2B cells expressing EV and DCBLD1 overexpression were suspended in a melted top layer containing 0.35% agar in complete DMEM. After solidification, 200 μL of complete DMEM was added to each well at a density of 100 cells per well. Soft-agar colonies were measured on day 28. For LUAD cells, treatment groups were seeded directly into 6-well plates at 100 cells per well and cultured for 14 days before crystal violet staining.

### Histological analysis

Tissue and organoid specimens were subjected to standard hematoxylin and eosin (H&E) staining following paraffin embedding, dewaxing, and rehydrating. For IHC staining, antigen retrieval was performed using citrate buffer (Servicebio, Wuhan, China), followed by blocking with 5% BSA for 1 h. The sections were then incubated with primary antibodies (Table S2) at 4 °C overnight. Afterwards, they were incubated with secondary antibodies (Table S2) for 1 h, treated with 3,3'-diaminobenzidine (Servicebio, Wuhan, China) and counterstained with hematoxylin (Sangon Biotech, Shanghai, China). Finally, the sections were dehydrated, and sealed in routine processing.

### Immunofluorescence (IF) analysis

Cells or organoids were seeded on coverslips, fixed with 4% paraformaldehyde (Sangon Biotech, Shanghai, China) for 15 min, permeabilized with 0.2% Triton X-100 (Sangon Biotech, Shanghai, China) for 10 min and blocked with 5% BSA (Sangon Biotech, Shanghai, China) for 1 h. They were then stained with primary antibodies (Table S2) overnight at 4 °C. The next day, cells were incubated with the corresponding fluorescent secondary antibodies (Table S2) at room temperature for 1 h. After washing with PBS, the cells were stained with DAPI (Yeasen, Shanghai, China) for 5 min. Fluorescence images were captured using a laser scanning confocal microscope (Leica, Wetzlar, Germany).

### RT-qPCR

Total RNA extraction was performed using TRIzol regent (Thermo Fisher Scientific, Waltham, MA, USA) and then reversely transcribed into cDNA using a reverse transcription kit (Accurate Biotechnology, ChangSha, China). Real-time PCR reaction was conducted on a LightCycler 480 System (Roche, Basel, Switzerland) using SYBR Green Pro Taq HS Premix (Accurate Biotechnology, ChangSha, China) according to the manufacturer's instructions. Primer sequences are listed in Table S1.

### RNA sequencing

A549 with the treatment of shCTRL and shDCBLD1 were collected for RNA sequencing. After RNA extraction, all downstream quality control steps, library preparation, sequencing was performed by GENEWIZ Co. Ltd (Suzhou, Jiangsu, China). DEGs analysis was performed using DESeq2. DEGs with |log2FC| > 1 and q value ≤ 0.05 were considered to be significantly differentially expressed. KEGG pathway analysis was then conducted to identify enriched biological pathways.

### Western blot

Proteins were extracted using RIPA lysis buffer (Yeasen, Shanghai, China) containing protease and phosphatase inhibitors (Cwbio, Shanghai, China). Protein concentrations were determined by BCA protein assay kit (Yeasen, Shanghai, China). 20 μg of protein per lane were separated by SDS-PAGE and transferred to PVDF membranes (Millipore, Burlington, MA, USA). The membranes were blocked in PBST with 5% non-fat milk for 1 h at room temperature, then incubated with primary antibodies (Table S2) overnight at 4 °C, followed by incubation with species matched secondary antibodies (Table S2) for 1 hour at room temperature. Signals were visualized by ECL KIT (Yeasen, Shanghai, China) using Bio-Rad ChemiDoc^TM^ MP system (Bio-Rad, USA).

### Immunoprecipitation (IP)

For exogenous interaction studies, HEK-293T cells in 6-cm dishes were transfected with 3 μg of each plasmid using Lipofectamine 3000 (Invitrogen, Carlsbad, USA). After 48 h transfection, cells were harvested and lysed in WB/IP lysis buffer (Yeasen, Shanghai, China) supplemented with protease inhibitor. For endogenous interaction studies, A549 and H1299 cells grown in 10 cm dishes were directly lysed using the same lysis buffer. In all IP experiments, 10% of the total cell lysate was used as the Input sample, with the remaining 90% used for the IP reaction. For exogenous interactions, lysates were incubated with anti-Flag or anti-HA beads (AlpaLifeBio, Shenzhen, China) overnight at 4 °C with gentle rotation. For endogenous interactions, lysates were first incubated with 2 μg of primary antibodies (anti-EGFR, anti-DCBLD1, or normal IgG control) overnight at 4 °C, followed by capture with Protein A/G beads (Epizyme, Shanghai, China) for an additional overnight incubation at 4 °C. Immunocomplexes were washed five times with ice-cold lysis buffer, then eluted by boiling in 30 μL of 2× SDS loading buffer at 100 °C for 10 min. The eluted proteins were subsequently analyzed by Western blot.

### EdU assay

PC9 cells were treated with or without gefitinib (0.5 μM) for 24 hours. The cells were then incubated with 10 μM EdU for 2 hours at 37 °C, then fixed with 4% paraformaldehyde for 15 minutes and permeabilized with 0.5% Triton X-100 for 10 min. EdU detection was performed using Click reaction containing Alexa Fluor 555 azide for 30 min at room temperature in the dark, followed by Hoechst 33342 counterstaining for 5 min. Fluorescent images were captured using a fluorescence microscope.

### Animal experiments

All animal experiments were conducted under protocols approved by the Shanghai Tongren Hospital's Experimental Animal Ethics Committee (Ethics Code 2023-105). All experimental mice were housed at the Laboratory Animal Center of Hongqiao International Institute of Medicine. Animals were maintained under standard laboratory conditions.

For xenograft studies, 2×10^6^ BEAS-2B stable expressing cells treated with B[a]P were subcutaneously injected into the right flank of BALB/c nude mice. Tumor growth was monitored triweekly. Harvested xenografts underwent IHC analyses.

For the induction of lung cancer, urethane (Sigma, St. Louis, MO, USA) was administered at a dose of 800mg/kg twice a week for a total of 10 injections. Mice were then maintained for 30 weeks before being sacrificed. Lungs were collected for tumor quantification and histological analyses.

For the orthotopic lung injection, 1×10^6^ PC9-Luc and PC9-Luc-DCBLD1 were suspended in 100 μL of a 1:1 mixture of Matrigel and PBS, then injected into the left lung of the mice. At day 12, gefitinib (50 mg/mL) was administered three times a week via oral gavage. Bioluminescent signals were acquired and recorded using an *in vivo* imaging system after mice were anesthetized with isoflurane.

To generate PDX models, tumor tissues from EGFR WT or EGFR Mut LUAD patients were dissected into 2 mm^3^ pieces and subcutaneously implanted into the interscapular region of SCID mice (F1). When tumors reached 1000 mm^3^, they were harvested and re-implanted into nude mice (F2). Following the same procedure, the tumors were propagated to F3 generation. Upon reaching 100 mm^3^, F3 tumors received intratumoral injections of control (shCTRL) or DCBLD1-targeting (shDCBLD1) lentiviral constructs at 5-day intervals.

### Statistical analysis

Data are presented as mean ± SD from three independent experiments. For comparisons between two groups, statistical significance was assessed using a two-tailed Student's t-test. For multiple group comparisons, one-way ANOVA was employed. ^*^p < 0.05, ^**^p < 0.01, ^***^p < 0.001. For survival analysis, Kaplan-Meier survival curves were compared using the log-rank test. All statistical analyses were conducted using GraphPad Prism 9.0 (GraphPad Software, San Diego, CA, USA).

## Results

### DCBLD1 overexpression drives proliferation and oncogenic transformation

**Figure 1:**
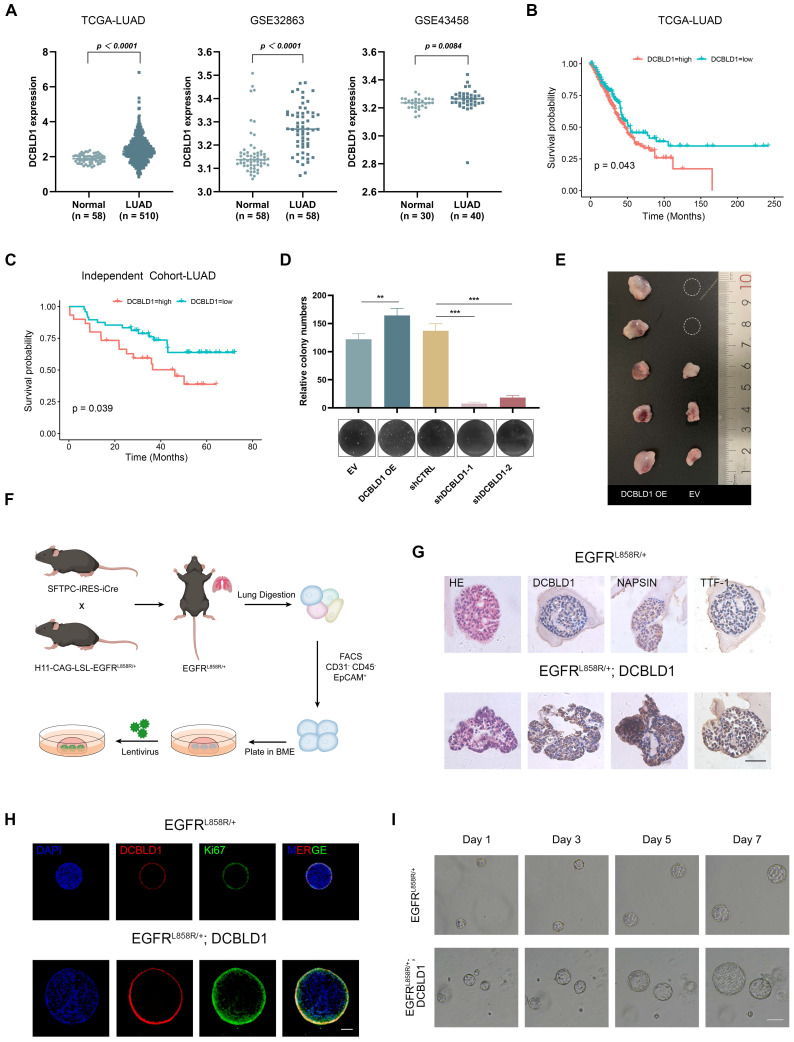
DCBLD1 overexpression promotes the proliferation and malignant transformation of both bronchial epithelial cells and lung organoids. **A**. Analysis of DCBLD1 mRNA expression in LUAD tissues compared to adjacent normal lung tissues using TCGA and GEO datasets. **B**-**C**. Overall survival analysis of LUAD patients stratified by high and low DCBLD1 mRNA expression levels from TCGA datasets (**B**) and an independent cohort (**C**). **D**. Soft-agar colony formation assay in BEAS-2B cells following DCBLD1 overexpression or knockdown. **E**. Representative images of excised tumors from mice subcutaneously implanted with BEAS-2B cells stably expressing DCBLD1 or empty vector control. **F**. Schematic illustration for establishing lung organoids derived from EGFR^L858R/+^ alveolar type 2 cells. **G**. Representative images of H&E and IHC staining of DCBLD1, NAPSIN A and TTF-1 in organoids under the indicated treatments. Scale bars, 50 μm. **H**. Immunofluorescence analysis of organoids stained for DCBLD1 (red), Ki67 (green), and nuclei with DAPI (blue). Scale bars, 100 μm. **I**. Representative bright-field images depicting the growth dynamics of organoids with the specified genotypes over a 7-day time course. Scale bars, 100 μm. ^**^p < 0.01, ^***^p < 0.001.

To elucidate the function of DCBLD1 in NSCLC, we initially examined its expression patterns across multiple datasets. Analysis of the TCGA-LUAD dataset, along with two additional public datasets (GSE32863 and GSE43458), consistently revealed significant upregulation of DCBLD1 in LUAD tissues compared to normal tissues (Figure 1A). Notably, subsequent survival analysis of the TCGA-LUAD cohort revealed that elevated DCBLD1 expression was significantly associated with poor clinical outcomes (Figure 1B). This finding was validated in our LUAD cohort (n = 78), where 5-year follow-up data demonstrated shorter survival times in patients with high DCBLD1 expression (Figure 1C).

Given these clinical findings, we investigated DCBLD1's function in NSCLC pathogenesis. We overexpressed and knocked down DCBLD1 in BEAS-2B cells using an expression vector and two distinct shRNAs, respectively. Compared to the empty vector (EV) control, DCBLD1 overexpression (OE) significantly enhanced cell viability, while knockdown reduced cellular growth rates (Figure S1A-B). Furthermore, soft agar colony formation assays demonstrated that DCBLD1 overexpression markedly increased transformation potential, while its knockdown impaired this capacity (Figure 1D).

To validate these *in vitro* findings *in vivo*, BEAS-2B cells were chemically transformed with benzo[a]pyrene (B[a]P) to induce malignant potential, followed by subcutaneous injection into nude BALB/c mice. While 60% of mice in the EV group developed tumors within two months, the DCBLD1 OE group exhibited a striking increase in tumorigenesis, with 100% of mice forming tumors (Figure 1E). Moreover, tumors in the DCBLD1 OE group demonstrated significantly greater weight (Figure S1C) and volume (Figure S1D). These data revealed that DCBLD1 overexpression enhances cell viability and transformation, leading to increased tumor formation in nude mice.

To elucidate the role of DCBLD1 in LUAD oncogenesis, we generated organoids from mouse AT2 cells, which have been proposed as a potential cell of origin for LUAD. EGFR^LSL-L858R^ mice were crossed with SFTPC-Cre mice to generate EGFR^L858R/+^ offspring. Genotyping of tail DNA with gel electrophoresis, followed by Sanger sequencing of lung tissue, confirmed the successful introduction of the heterozygous EGFR^L858R^ mutation in lung tissue (Figure S2A-B). Following tissue dissociation and immunostaining, FACS was used to isolate CD45^-^, CD31^-^, EPCAM^+^ cells, which were then expanded to form organoids. These organoids were infected with either lentiviral EV or DCBLD1 OE, and protein levels were verified by western blotting (Figure 1F, Figure S2C). H&E staining revealed that both organoid groups displayed characteristic acinar or large glandular morphologies. Notably, EGFR^L858R/+^; DCBLD1 organoids maintained elevated DCBLD1 expression and expressed key LUAD markers, including NAPSIN A and TTF-1 (Figure 1G). To assess the proliferative advantage conferred by DCBLD1 overexpression, we performed IF staining for the proliferation marker Ki67. Compared to parental organoids, EGFR^L858R/+^; DCBLD1 organoids exhibited significantly higher Ki67 expression (Figure 1H, Figure S1E). Moreover, they consistently formed larger organoids (Figure 1I, Figure S1F-G). These data indicate that DCBLD1 overexpression promotes malignant transformation and overgrowth in oncogenic EGFR-driven LUAD organoids.

### The DCBLD1 depletion suppresses the initiation of LUAD

**Figure 2:**
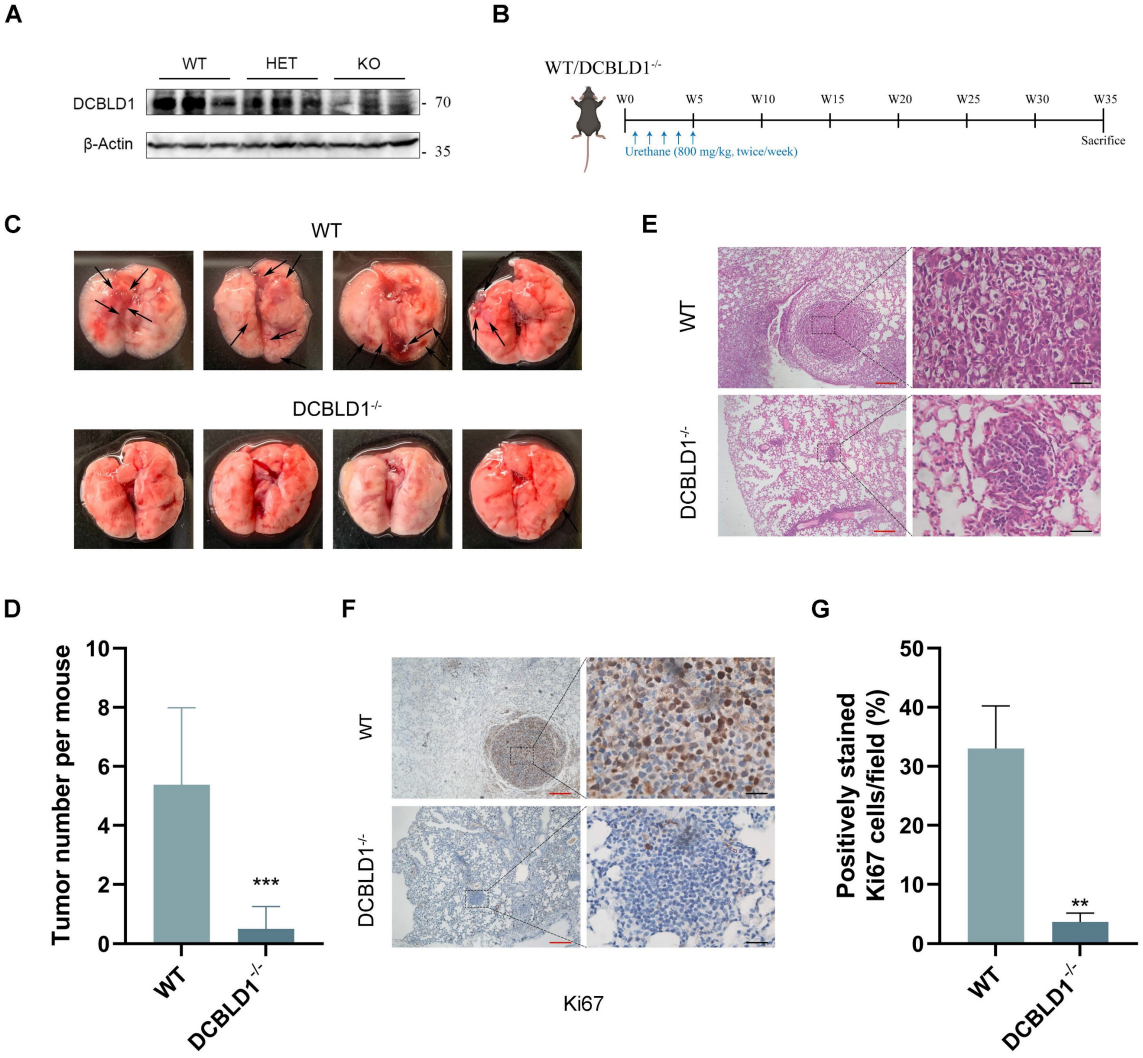
DCBLD1 deficiency attenuates the initiation and progression of LUAD. **A**. Western blot analysis confirming DCBLD1 protein deletion in DCBLD1^-/-^mice compared to wild-type (WT) controls. **B**. Schematic illustration of the experimental timeline for urethane-induced LUAD development in WT and DCBLD1^-/-^ mice. **C**. Representative gross morphology of lung specimens from WT and DCBLD1^-/-^ mice after 30 weeks of urethane treatment. Black arrows indicate visible tumor lesions. **D**. Quantification of tumor nodules per lung in WT and DCBLD1^-/-^ mice. **E**-**G**. Lung tissue sections from WT and DCBLD1^-/-^ mice were analyzed by H&E staining (**E**) and IHC staining for Ki67 (**F**), with quantitative analysis of Ki67-positive cells (**G**). Red scale bars: 400 μm, Black scale bars:40 μm. ^**^p < 0.01, ^***^p < 0.001.

To further elucidate the role of DCBLD1 in LUAD oncogenesis, a DCBLD1 knockout (KO, DCBLD1^-/-^) mouse model was established. The successful deletion of DCBLD1 was verified through tail DNA genotyping via gel electrophoresis and further confirmed by tissue mRNA and protein expression analyses (Figure 2A, Figure S3A-C). To induce LUAD, both WT and DCBLD1^-/-^ mice received urethane (800 mg/kg) twice a week for a total of 10 treatments, followed by sacrifice at 35 weeks (Figure 2B). Strikingly, DCBLD1^-/-^ mice demonstrated marked resistance to LUAD development, with tumors occurring in only 25% (2/8) of DCBLD1^-/-^ mice compared to 87.5% (7/8) of WT controls over the 35-week period (Figure 2C). Additionally, body weights were comparable between the groups (Figure S3D). Quantitative assessment revealed markedly reduced tumor burden in DCBLD1^-/-^ mice relative to WT counterparts (Figure 2D). H&E staining of lungs demonstrated that minimal alveolar lesions in DCBLD1^-/-^ mice, whereas WT mice developed extensive adenocarcinomas (Figure 2E). IHC staining confirmed the absence of DCBLD1 in KO mouse pulmonary lesions and demonstrated decreased Ki-67 expression, indicating reduced proliferative activity compared to WT controls (Figure 2F-G, Figure S3E). These results demonstrate that DCBLD1 deletion suppresses lung tumorigenesis, highlighting its essential role in LUAD development.

### DCBLD1 positively modulates the EGFR signaling pathway

**Figure 3:**
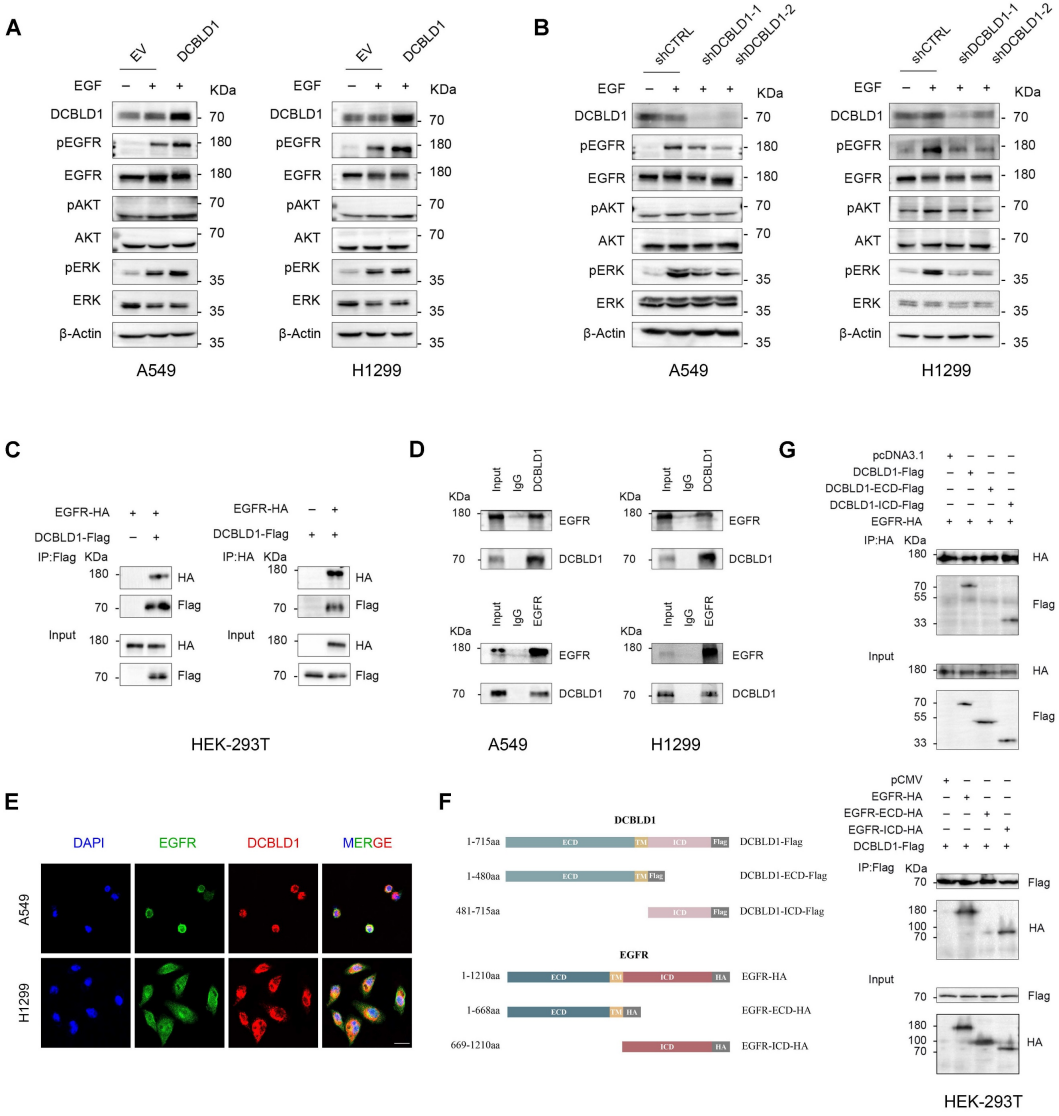
DCBLD1 modulates EGFR signaling and physically interacts with EGFR. **A**-**B.** Western blot analysis of the EGFR signaling pathway in A549 and H1299 cells upon DCBLD1 overexpression (**A**) or knockdown (**B**). **C.** Co-immunoprecipitation analysis of EGFR-DCBLD1 interaction in HEK-293T cells. Cells were transfected with EGFR-HA and/or DCBLD1-Flag plasmids for 48 h. Immunoprecipitation was performed using Flag or HA antibodies, followed by Western blot analysis. **D.** Endogenous co-immunoprecipitation assays were performed in A549 and H1299 cells using IgG control, DCBLD1 or EGFR antibodies. Immunoprecipitated complexes were analyzed by Western blot with EGFR and DCBLD1 antibodies. **E.** Representative confocal microscopy images showing co-localization of EGFR (green) and DCBLD1 (red) in A549 and H1299 cells. Scale bar, 20 μm. **F.** Schematic illustration of full-length DCBLD1 and EGFR proteins, and their corresponding truncation mutants. **G.** HEK-293T cells were co-transfected with EGFR-HA and either pcDNA3.1 vector, DCBLD1-Flag, DCBLD1-ECD-Flag, or DCBLD1-ICD-Flag (upper panel). Alternatively, cells were co-transfected with DCBLD1-Flag and either pCMV vector, EGFR-HA, EGFR-ECD-HA, or EGFR-ICD-HA (lower panel). Co-immunoprecipitation assays were performed using HA or Flag antibodies, followed by Western blot analysis to identify interaction domains.

DCBLD1 amino acid sequences are highly conserved across vertebrates, and its intracellular region contains several tyrosine phosphorylation sites. To investigate whether intracellular phosphorylation was an important regulator of DCBLD1 biological function, we transfected HEK-293T with Flag-tagged DCBLD1 or HA-tagged EGFR. Using the 4G10 antibody that recognizes phosphorylated tyrosine residues, we performed IP and detected phosphorylated EGFR as a positive control, while no tyrosine phosphorylation signal was observed for DCBLD1 (Figure S4A). Given that DCBLD1 and DCBLD2 are transmembrane proteins belonging to the same family, we hypothesized that they might form heterodimeric complexes to exert their functional roles. However, Co-IP experiments showed no interaction between these proteins (Figure S4B). To elucidate the oncogenic mechanism of DCBLD1, we performed RNA sequencing on A549 cells with DCBLD1 knockdown (shDCBLD1) and control cells (shCTRL), which revealed 264 differentially expressed genes (DEGs) (Figure S4C). KEGG pathway analysis of DEGs revealed significant enrichment of MAPK signaling pathway (Figure S4D). Additionally, TCGA LUAD revealed that DCBLD1-correlated genes were enriched in MAPK and PI3K/AKT signaling pathways (Figure S4E). Previous studies have shown that DCBLD family proteins interact with EGFR signaling. Feng et al. demonstrated that DCBLD2 functions as a signal relay for oncogenic EGFR signaling 18, while Chen et al. showed that CD146 protects DCBLD2 from degradation, thereby activating the PI3K/AKT pathway 19. Consistently, our earlier work revealed stronger associations between DCBLD1 SNPs and LUAD susceptibility in EGFR mutation-positive cases compared to negative cases 20. These collective findings led us to hypothesize that DCBLD1 promotes tumor growth through EGFR signaling modulation. qPCR and immunoblot analyses demonstrated that DCBLD1 overexpression or knockdown in A549 and H1299 cell lines did not alter EGFR mRNA or protein levels (Figure 3A-B, Figure S4F-G). Notably, DCBLD1 overexpression enhanced EGF-induced phosphorylation of EGFR and its downstream effectors ERK and AKT, whereas its silencing attenuated their phosphorylation (Figure 3A-B, Figure S5A-D). Furthermore, IHC staining revealed elevated pEGFR levels in DCBLD1-overexpressing BEAS-2B subcutaneous xenografts compared to EV controls (Figure S5E), while DCBLD1 KO mice showed reduced pEGFR levels in lung nodules relative to WT controls (Figure S5F). These findings establish DCBLD1 as a positive regulator of EGFR activation and its downstream signaling pathways.

### DCBLD1 interacts with EGFR through their intracellular domains

Based on our findings that DCBLD1 modulates EGFR signaling activities and phosphorylation status, we hypothesized that DCBLD1 might physically interact with EGFR to regulate its function. To test this hypothesis, we first validated the DCBLD1-EGFR interaction by Co-IP using HEK-293T cells exogenously expressing DCBLD1-Flag and EGFR-HA (Figure 3C). The interaction was further confirmed at the endogenous level in both A549 and H1299 cells (Figure 3D). Additionally, immunofluorescence analysis revealed DCBLD1-EGFR colocalization, supporting their physical association (Figure 3E).

To map the interaction domains between DCBLD1 and EGFR, we generated two truncations of each protein (Figure 3F). Co-IP analysis of HEK-293T cells expressing EGFR with either empty vector, full-length DCBLD1, DCBLD1 extracellular domain (ECD), or DCBLD1 intracellular domain (ICD) demonstrated that the interaction required the DCBLD1 ICD segment (Figure 3G). Similarly, cells expressing DCBLD1 with either empty vector, full-length EGFR, EGFR-ECD, or EGFR-ICD showed that the interaction depended on EGFR ICD segment (Figure 3G). Together, these results establish that DCBLD1 and EGFR interact through their respective intracellular domains.

### DCBLD1 suppresses the PTP1B-mediated dephosphorylation of EGFR

**Figure 4:**
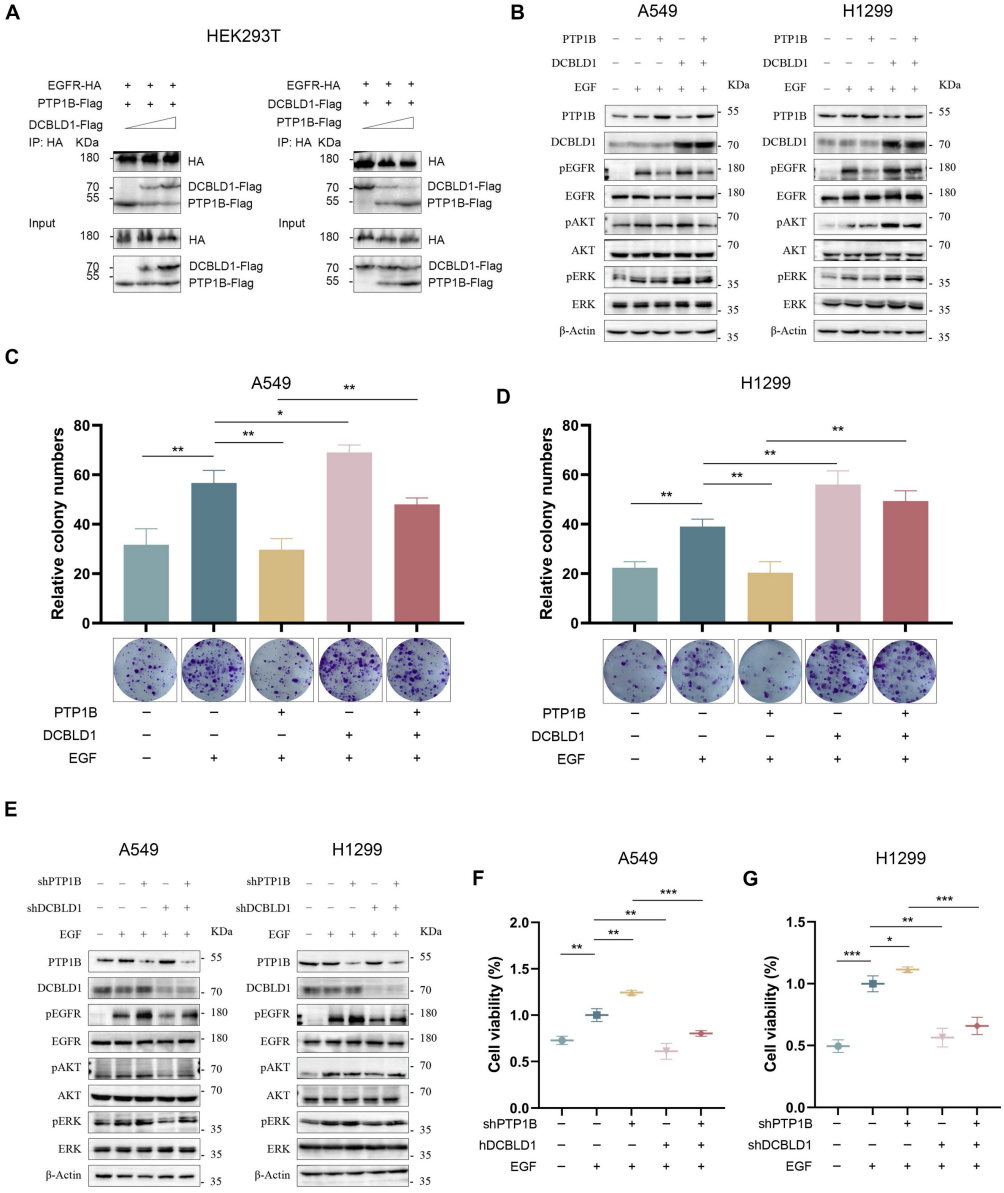
DCBLD1 promotes LUAD growth by interfering with PTP1B-mediated EGFR dephosphorylation. **A.** HEK293T cells were co-transfected with EGFR-HA, PTP1B-Flag, and increasing concentrations of DCBLD1-Flag (left panel). Alternatively, cells were co-transfected with EGFR-HA, DCBLD1-Flag, and increasing concentrations of PTP1B-Flag (right panel). Co-immunoprecipitation assays were performed using HA antibodies. Immunoprecipitated complexes were analyzed by Western blot with HA and Flag antibodies. **B**-**D**. LUAD cells expressing DCBLD1, PTP1B, both or neither were treated with or without 100 ng/mL EGF for 15 min. Protein levels were analyzed by Western blot analyses (**B**) and long-term proliferative capacity was evaluated by colony formation assays in A549 (**C**) and H1299 (**D**). **E**-**G**. LUAD cells expressing shDCBLD1, shPTP1B, both or neither were treated with or without 100 ng/mL EGF for 15 min. Protein levels were analyzed by Western blot analyses (**E**) and cell viability was assays by CCK-8 assays in A549 (**F**) and H1299 (**G**). ^*^p < 0.05, ^**^p < 0.01, ^***^p < 0.001.

The phosphorylation state of EGFR can be modulated through three primary mechanisms: ligand-induced autoactivation, transactivation by other receptors or kinases, and dephosphorylation by intracellular phosphatases 21-23. Since DCBLD1 lacks kinase activity, we investigated its potential influence on phosphatases. Previous studies have established that protein tyrosine phosphatase 1B (PTP1B) is the primary phosphatase that negatively regulates EGFR signaling 24-26. Analysis of TCGA LUAD dataset revealed significantly decreased PTP1B mRNA levels in LUAD tissues compared to adjacent normal tissues (Figure S6A). Additionally, Lei et al. have shown that DCBLD2 modulates VEGFR-2 phosphorylation by affecting its interaction with PTP1B 27. These findings led us to hypothesize that DCBLD1 might enhance EGFR phosphorylation by either modulating PTP1B activity or disrupting EGFR-PTP1B interaction. Both qPCR and immunoblot analyses revealed that DCBLD1 did not affect PTP1B expression levels (Figure 4B and E, Figure S6B-C).

Intriguingly, we found that DCBLD1 impaired the association between PTP1B and EGFR in a dose-dependent manner. Conversely, increasing concentrations of PTP1B progressively diminished the interaction between DCBLD1 and EGFR (Figure 4A). This interference pattern strongly suggests a competitive binding mechanism, whereby DCBLD1 and PTP1B compete for overlapping or adjacent binding sites on EGFR.

To further validate this mechanism, we investigated whether DCBLD1 could modulate PTP1B-mediated effects on EGFR signaling. As shown in Figure 4B and Figure S6D-E, PTP1B overexpression suppressed EGFR phosphorylation but had minimal effects on downstream AKT and ERK phosphorylation in the absence of DCBLD1. Intriguingly, when DCBLD1 was co-expressed, PTP1B-mediated inhibition significantly extended to both AKT and ERK phosphorylation. Furthermore, DCBLD1 overexpression effectively rescued PTP1B-induced suppression of cell viability and colony formation (Figure 4C-D, Figure S6F-G). Conversely, as shown in Figure 4E and Figure S6H-I, PTP1B depletion enhanced EGFR phosphorylation but had modest effects on AKT and ERK phosphorylation under normal conditions. However, in DCBLD1-knockdown cells, where the competitive protection against PTP1B is lost, PTP1B depletion resulted in activation of both AKT and ERK phosphorylation. Consistently, the reduction in cell viability and colony formation capacity induced by DCBLD1 knockdown was effectively rescued by concurrent PTP1B knockdown. (Figure 4F-G, Figure S6J-K). These results collectively suggest that DCBLD1 acts as a phosphorylation protector that enhances the responsiveness of downstream signaling cascades to changes in EGFR phosphorylation status. By competitively binding EGFR and blocking PTP1B-mediated dephosphorylation, DCBLD1 exerts a dominant role in regulating oncogenic signaling outputs.

### Overexpression of DCBLD1 confers resistance to EGFR TKI both *in vitro* and *in vivo*

**Figure 5:**
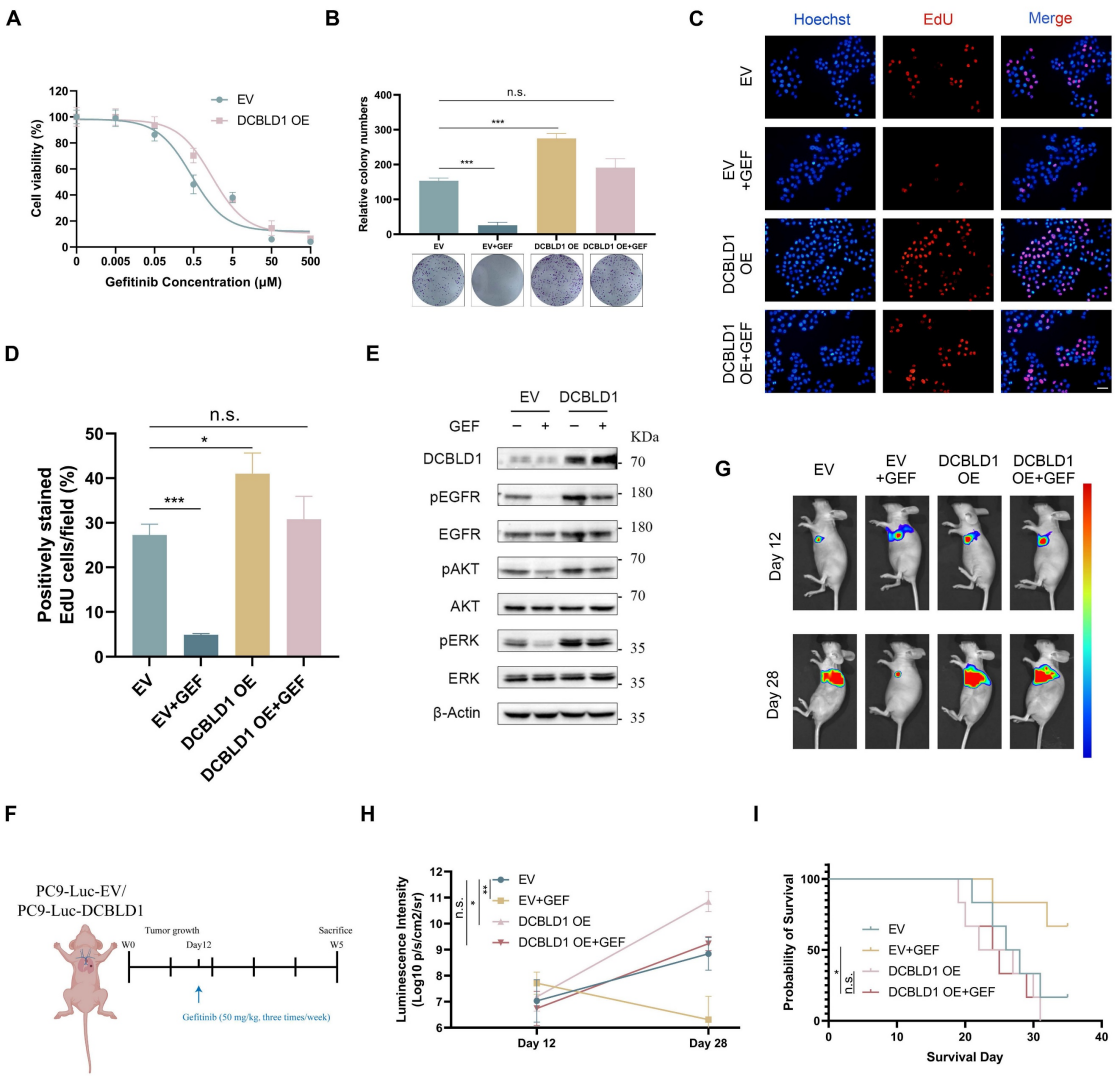
DCBLD1 overexpression confers resistance to EGFR TKI treatment both *in vitro* and *in vivo*. **A**. Dose-response curves of PC9 cells expressing empty vector (EV) or DCBLD1 overexpression (OE) treated with gefitinib (GEF). **B**. Representative images and quantification of colony formation in EV or DCBLD1 OE cells with or without GEF treatment. **C**-**D.** Representative images of EdU assay (**C**) showing proliferating cells (red) and Hoechst (blue), and quantification (**D**) of EdU-positive cells in EV or DCBLD1 OE cells with or without GEF treatment. Scale bar, 100 μm. **E**. Western blot analysis of indicated proteins in EV and DCBLD1 OE cells treated with or without GEF treatment. **F**. Schematic illustration of the experimental design for orthotopic xenograft model. **G**. Bioluminescence imaging of orthotopic lung tumors derived from luciferase-expressing PC9-EV or PC9-DCBLD1 cells at specific timepoints with indicated treatment. **H**. Quantification of bioluminescence intensity over time (n=5). **I**. Kaplan-Meier survival curve of the indicated orthotopic xenograft models with or without GEF treatment (n=6). n.s., not significant;^ *^p < 0.05, ^**^p < 0.01, ^***^p < 0.001.

EGFR TKIs have emerged as the standard first-line therapy for advanced LUAD patients with activating EGFR mutations, demonstrating superior response rates and benefits in progression-free survival compared to conventional chemotherapy 28,29. Despite the initial efficacy of TKIs, the constitutive activation of EGFR and its downstream signaling cascades can attenuate therapeutic sensitivity in lung cancer 30,31. Given the correlation between DCBLD1 overexpression and increased phosphorylation of EGFR, ERK and AKT, we investigated whether DCBLD1 overexpression contributes to TKI resistance in LUAD. We utilized PC9 cells harboring an EGFR exon 19 deletion mutation, which typically exhibits high sensitivity to gefitinib (GEF). As shown in Figure 5A, DCBLD1 OE increased the IC50 values of GEF from 0.47 μM to 1.57 μM. Moreover, while GEF treatment entirely suppressed cell colony formation in the EV group, it had no such effect on the DCBLD1 OE group, which maintained a level of colony formation comparable to the untreated EV group (Figure 5B). We also evaluated the effects of GEF on cellular proliferation *in vitro* using a EdU incorporation assay. The results showed that GEF nearly suppressed proliferation in the EV group, whereas the DCBLD1 OE group exhibited significant resistance to this inhibitory effect. (Figure 5C-D). As expected, with the treatment of GEF for 24 h, EV group caused significant decreases in EGFR, ERK and AKT phosphorylation relative to untreated cells. However, in DCBLD1 OE cells, GEF effects were minimal, causing only a slight decrease in EGFR, ERK and AKT phosphorylation (Figure 5E). These results indicated that DCBLD1 overexpression attenuates GEF-induced inhibition of EGFR phosphorylation and downstream signaling pathways.

To further investigate the effects of inactivation of DCBLD1 to GEF *in vivo*, luciferase (luc)-labeled DCBLD1 OE cells and EV cells were injected into the left lung of mice. The mice were randomly divided into four groups and treated with or without GEF (Figure 5F). Bioluminescence images revealed that tumor xenografts grew remarkably larger in the DCBLD1 OE group than in the EV group. Although tumor growth was partially limited by GEF in DCBLD1 OE mice, this tendency towards increased growth was not reversed (Figure 5G-H). In addition, DCBLD1 OE mice treated with GEF exhibited shorter survival times than EV mice treated with GEF (Figure 5I). Based on the evidence presented, we believe that DCBLD1 overexpression leads to resistance to TKI therapy in LUAD.

### Targeting DCBLD1 as a therapeutic opportunity for LUAD regardless of EGFR mutation status

**Figure 6:**
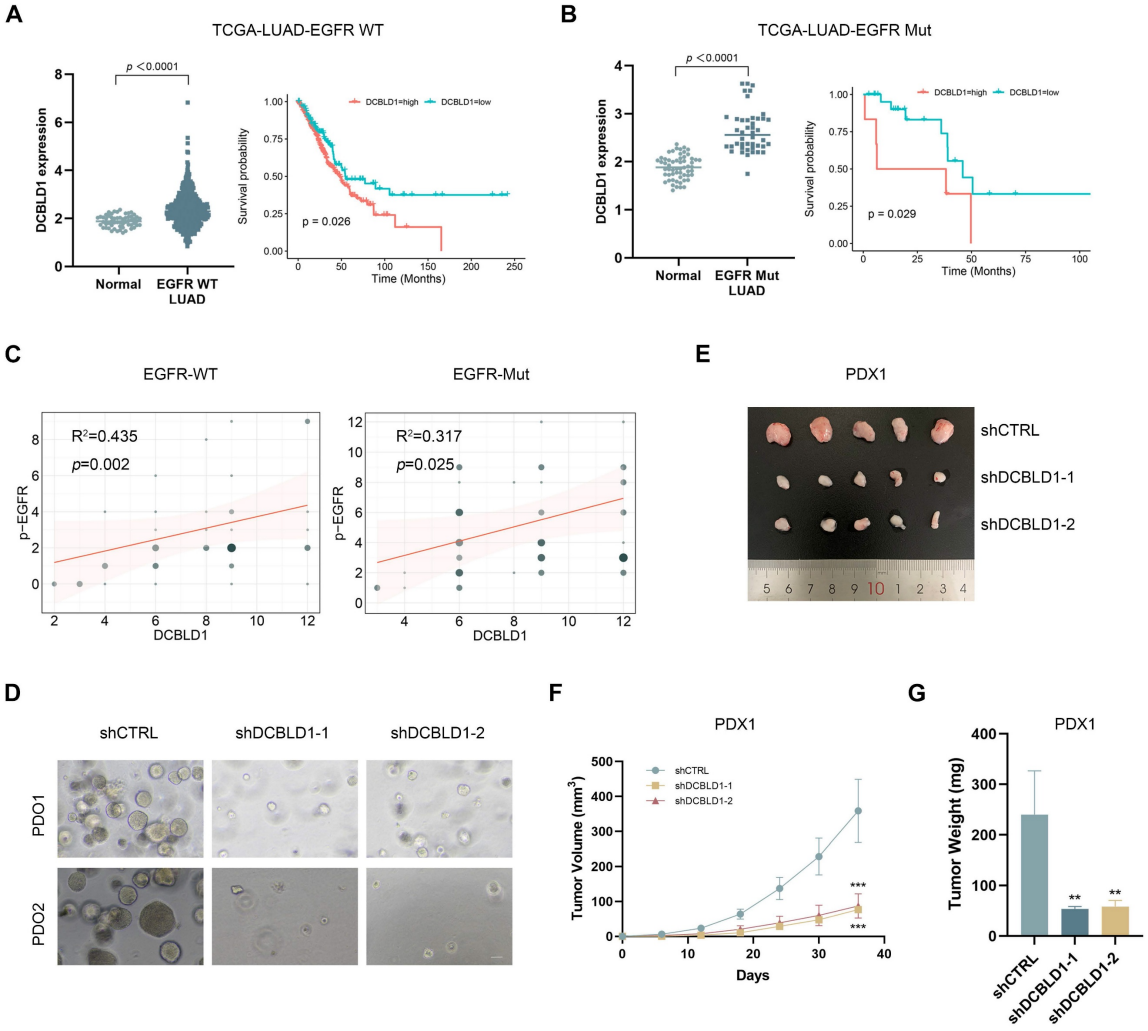
DCBLD1 knockdown inhibits tumor growth in LUAD PDOs and PDXs, regardless of EGFR mutation status. **A**-**B.** DCBLD1 mRNA expression in EGFR wild type (WT; **A**) and EGFR mutant (Mut; **B**) LUAD versus normal tissues, with corresponding overall survival analysis based on DCBLD1 expression levels in each subgroup. **C.** Correlation between DCBLD1 and pEGFR expression in LUAD samples with EGFR WT or Mut status. **D**. Representative bright-field images of LUAD patient-derived organoids (PDO) following DCBLD1 knockdown. Scale bars, 50 μm. **E**. Gross morphological images of patient-derived xenograft (PDX) 1 treated with shCTRL or shDCBLD1. **F**-**G.** Relative tumor volume (F) and tumor weight (G) of PDX1 treated with shCTRL or shDCBLD1. Tumor volume was determined by caliper measurements using the formula (length × width²)/2. ^**^p < 0.01,^ ***^p < 0.001.

Given our findings regarding DCBLD1's regulatory role in the EGFR signaling pathway, we performed additional analysis of the TCGA-LUAD dataset stratified by EGFR mutation status. This analysis revealed significantly elevated DCBLD1 expression in both EGFR WT and EGFR Mut LUAD tissues compared to adjacent non-tumor tissues (Figure 6A). Furthermore, the association between elevated DCBLD1 expression and poor prognosis remained consistent across both EGFR-WT and EGFR-mutant subgroups, suggesting that the prognostic value of DCBLD1 is independent of EGFR mutation status (Figure 6B). Correlation analysis further demonstrated significant positive associations between DCBLD1 and pEGFR expression levels in both EGFR WT (R² = 0.435, *p* = 0.002) and EGFR Mut patients (R² = 0.317, *p* = 0.025) (Figure 6C), supporting our mechanistic findings that DCBLD1 enhances EGFR phosphorylation across different molecular subtypes.

Following IHC confirmation of DCBLD1 overexpression in two cases (Case 1: EGFR WT; Case 2: EGFR Mut, exon 19 deletion; Figure S7A), tumor tissues were used to establish both PDO and PDX models. In the PDOs model, DCBLD1 knockdown resulted in a significant reduction in organoid number and size, suggesting impaired tumor growth and survival *ex vivo* (Figure 6D, Figure S7B-C). Concurrently, in the PDXs model, DCBLD1 knockdown led to a marked decrease in tumor weight and tumor volume, demonstrating the efficacy of DCBLD1 inhibition *in vivo* (Figure 6E-G, Figure S7D-F). IHC staining demonstrated that DCBLD1 knockdown sharply decreased the phosphorylated EGFR, AKT, ERK and Ki67 (Figure S7G), consistent with our *in vitro* observations. Taken together, these data indicate the potential therapeutic benefits of targeting DCBLD1 in LUAD, independent of EGFR mutation status.

## Discussion

Building upon our previous identification of DCBLD1 as a lung cancer susceptibility gene, this study revealed that DCBLD1 actively participates in LUAD tumorigenesis rather than merely serving as a genetic susceptibility marker. To elucidate DCBLD1's role in lung carcinogenesis, we employed two complementary models. First, organoids derived from EGFR^L858R/+^ spontaneous lung cancer mouse-derived AT2 demonstrated a gain-of-function effect of DCBLD1 overexpression in promoting LUAD progression. Second, tumors induced in DCBLD1 KO mice using urethane established a loss-of-function paradigm, confirming the inhibitory effect of DCBLD1 deficiency on lung cancer initiation. These findings collectively identify DCBLD1 as a crucial mediator in LUAD development.

The DCBLD receptor family, comprising DCBLD1 and DCBLD2, represents a highly conserved group of vertebrate transmembrane proteins 32. While DCBLD2 has been extensively studied as a co-receptor for various receptor tyrosine kinases, including VEGFR, EGFR, PDGFR and INSR 18,27,33,34, the mechanism underlying DCBLD1 function remain comparatively unexplored. Recent study has implicated DCBLD1 in cancer progression, demonstrating its role in cervical cancer through lactylation-driven modulation of the pentose phosphate pathway 35. Our RNA-seq enrichment analysis identified significant involvement of PI3K/AKT and MAPK signaling pathways, both of which are critical downstream effectors of EGFR. Combined with our previous genetic studies showing stronger associations between DCBLD1 SNPs and LUAD susceptibility in EGFR mutation-positive cases, this evidence led us to hypothesize that DCBLD1 regulates EGFR signaling. Our mechanistic investigations revealed that DCBLD1 physically interact with EGFR to modulates its signaling activities and phosphorylation status.

**Figure 7:**
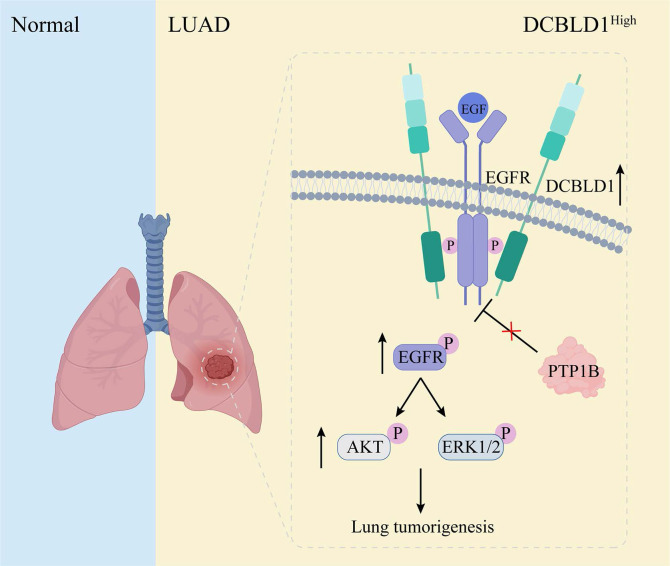
Schematic illustration of effects of DCBLD1 on LUAD tumorigenesis.

The dynamic regulation of EGFR phosphorylation levels is maintained through phosphorylation and dephosphorylation processes 36. Several phosphatases containing Src homology 2 and phosphotyrosine-binding domains mediate EGFR dephosphorylation and inactivation 37-39. However, the effects of these phosphatases on EGFR and its downstream signaling pathways are not uniformly inhibitory. Some phosphatases may dephosphorylate proteins that negatively regulate EGFR, paradoxically resulting in a positive regulation of EGFR signaling 40,41. Among the representative phosphatases of EGFR, PTP1B exhibits particularly complex regulatory patterns, functioning as both a tumor suppressor and promoter. Its ultimate effect on tumor progression is determined by the delicate balance of the pro- and anti-tumorigenic mechanisms, which are heavily influenced by the specific molecular profile of the cancer cells. For instance, in a tumor with hyperactive Src signaling, PTP1B can dephosphorylate the inhibitory tyrosine residue Tyr527 of Src kinase, thereby promoting tumor growth 42. Conversely, in EGFR-dependent tumors, PTP1B suppresses tumor growth by dephosphorylating EGFR upon EGF stimulation 43. In this study, we have uncovered that DCBLD1 forms a complex with EGFR, which effectively suppresses the PTP1B-mediated dephosphorylation of EGFR in the cytoplasm. This interaction results in sustained EGFR activation, subsequently driving LUAD tumorigenesis (Figure 7). Notably, PTP1B regulation of EGFR downstream signaling requires DCBLD1 expression, likely due to phosphatase redundancy and DCBLD1's role as a scaffolding protein that stabilizes EGFR-effector complexes.

Despite remarkable progress in EGFR-targeted therapies, substantial challenges persist in treating LUAD, which vary according to EGFR mutation status. For EGFR Mut LUAD patients, the emergence of drug-resistant EGFR mutations through tumor evolution and selective pressure from therapeutic interventions underscores the critical need for novel EGFR-targeting agents with alternative inhibitory mechanisms. The continuous adaptation of cancer cells to EGFR-targeted therapies represents a significant challenge in maintaining long-term treatment efficacy. This phenomenon highlights the dynamic nature of tumor biology and the necessity for innovative approaches to circumvent resistance mechanisms 44-47.

Recent advances in understanding resistance mechanisms have prompted a paradigm shift in anti-EGFR therapeutic development, extending beyond traditional kinase domain targeting to explore alternative regulatory mechanisms. A notable example is the development of amivantamab, a bispecific antibody targeting both EGFR and MET receptors. This approach addresses the complex interplay between these pathways, as MET activation can contribute to EGFR signaling through shared downstream effectors. Following successful validation in the CHRYSALIS, CHRYSALIS-2, and PAPILLON clinical trials, amivantamab received FDA approval for treating metastatic NSCLC harboring EGFR exon 20 insertion mutations 48,49. Transmembrane proteins DCBLD1, which also modulates EGFR signaling, present another potential avenue for innovative therapeutic targeting.

For EGFR WT LUAD patients, the situation is even more challenging as they derive limited benefit from EGFR TKIs due to the drugs' specific targeting of mutant EGFR kinase domain, despite the presence of active EGFR signaling through bypass pathways 50,51. Although conventional therapies such as chemotherapy and immunotherapy are available for these patients, the lack of effective targeted approaches represents a significant therapeutic gap.

We stratified the TCGA-LUAD dataset based on EGFR mutation status and observed significantly elevated DCBLD1 expression in both EGFR WT and EGFR Mut LUAD tissues compared to adjacent non-tumor tissues. Furthermore, elevated DCBLD1 expression correlated with poor survival outcomes across both EGFR WT and EGFR Mut patient. Additionally, significant positive correlations were observed between DCBLD1 and pEGFR expression levels in both EGFR WT and EGFR Mut subgroups. To address the therapeutic challenges posed by these subtypes, we investigated DCBLD1 as a potential therapeutic target using both *ex vivo* PDO and *in vivo* PDX models established from EGFR WT and EGFR Mut LUAD patients. The therapeutic efficacy of DCBLD1 inhibition was validated in these models, demonstrating its potential as a novel therapeutic strategy. These findings suggest that targeting DCBLD1 could provide a promising treatment option for both EGFR WT LUAD patients who currently lack targeted therapy options and EGFR Mut patients as an alternative therapeutic approach.

In summary, our study provides novel insights into the oncogenic role of DCBLD1 in LUAD. Mechanistic investigations revealed that DCBLD1 enhances EGFR signaling by preventing its interaction with the phosphatase PTP1B. Notably, DCBLD1 upregulation was observed in both EGFR WT and EGFR Mut LUAD patients, correlating with poor clinical outcomes. The therapeutic efficacy of DCBLD1 inhibition was validated in both PDO and PDX models, demonstrating significant antitumor effects independent of EGFR mutation status. Further research will focus on developing DCBLD1-targeted inhibitors and evaluating their efficacy, both as monotherapies and in combination with standard treatments, across diverse disease stages.

## Supplementary Material

Supplementary figures and tables.

## Abbreviations

AT2: alveolar type II
DCBLD1: discoidin, CUB and LCCL domain containing 1
ECD: extracellular domain
EV: empty vector
GEF: gefitinib
GWAS: genome-wide association studies
ICD: intracellular domain
IHC: immunohistochemistry
LUAD: Lung adenocarcinoma
NSCLC: Non-small cell lung cancer
OE: overexpression
PDO: patient-derived organoid
PDX: patient-derived xenograft
SNP: single nucleotide polymorphism
TKIs: tyrosine kinase inhibitors
WT: wild type
