# Supplementary material for: DCBLD1 Promotes Lung Tumorigenesis by Inhibiting PTP1B Dephosphorylation of EGFR
Source: Int J Biol Sci. 2026 Jan 1;22(2):684–700. doi: 10.7150/ijbs.112100 (PMC12781078; doi:10.7150/ijbs.112100)
Supplement: Supplementary file 1 — Supplementary figures and tables. [file ijbsv22p0684s1.pdf]

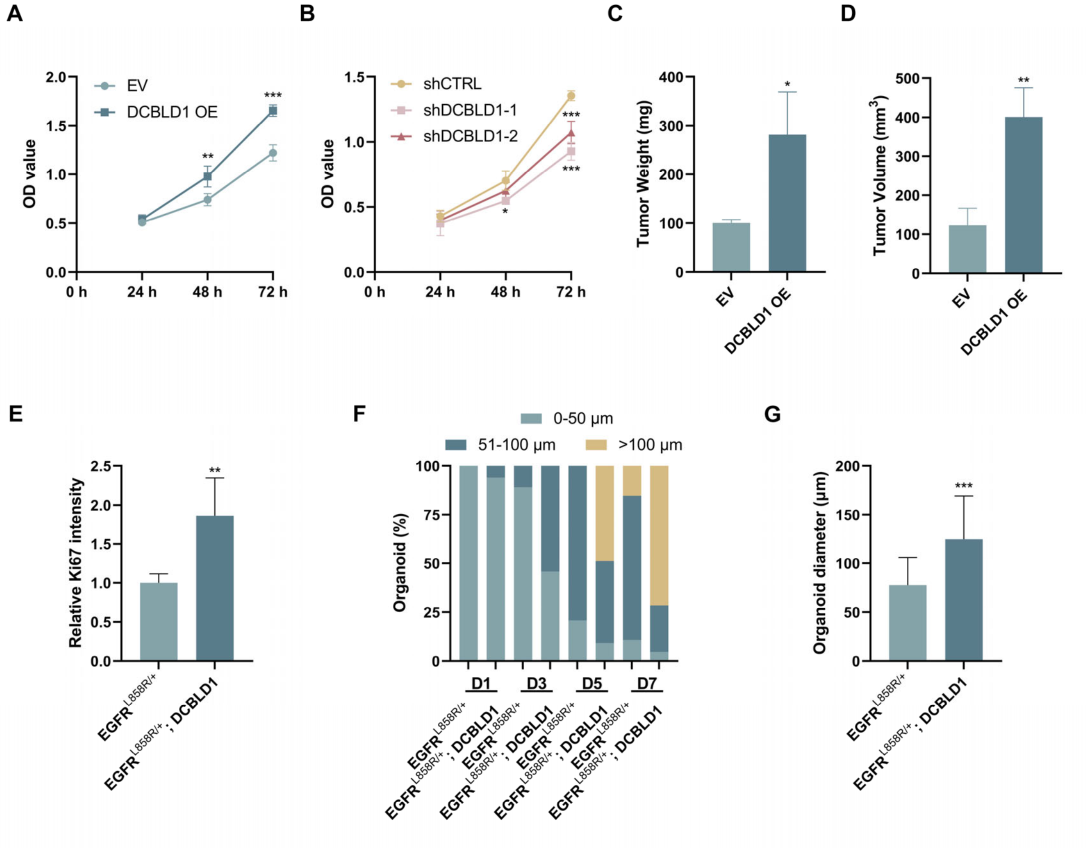

Supplementary Figure S1. DCBLD1 promotes cell viability, tumor growth, and organoid development. **A**-**B**. Cell viability measured by CCK-8 assay in BEAS-2B cells with stable DCBLD1 overexpression (**A**) or shRNA-mediated DCBLD1 knockdown (**B**). **C**-**D**. Quantification of tumor weight (**C**) and tumor volume (**D**) in nude mice bearing subcutaneous xenografts derived from DCBLD1-overexpressing or control BEAS-2B cells. **E**. Quantitative analysis of relative Ki67 intensity in organoids with specified genotypes. **F**-**G**. Quantitative analysis of organoid size distribution (**F**) and organoid diameter (**G**) with specified genotypes. $^{*}p < 0.05$, $^{**}p < 0.01$, $^{***}p < 0.001$.

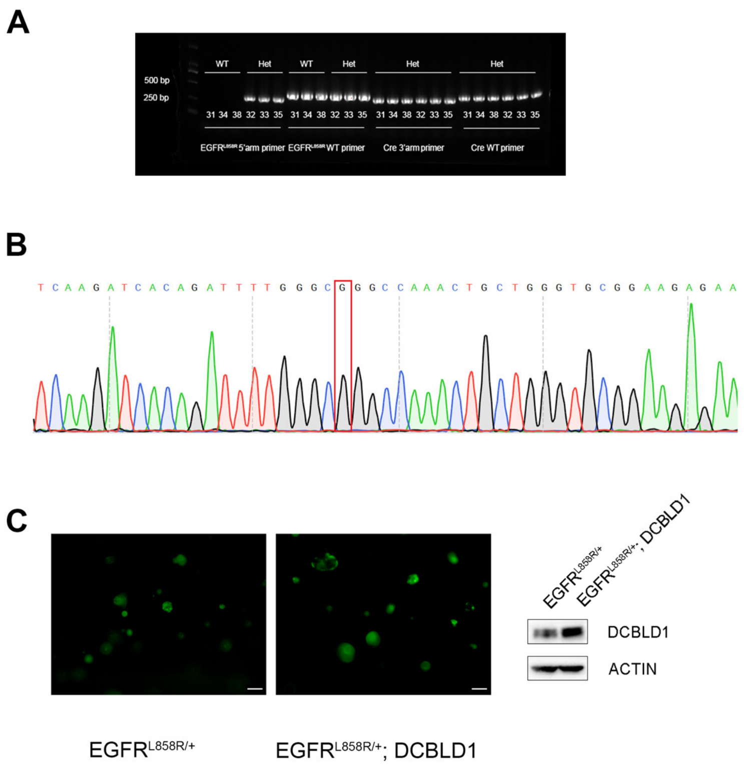

Supplementary Figure S2. Validation of EGFR$^{L858R/+}$ mouse model and DCBLD1 overexpression in organoids. **A-B**. Validation of EGFR^{L858R} transgenic mouse model using PCR-based genotyping (**A**) and Sanger sequencing (**B**). **C**. Western blot analysis confirming DCBLD1 overexpression in lung organoids following lentiviral transduction. Scale bar, 100 µm.

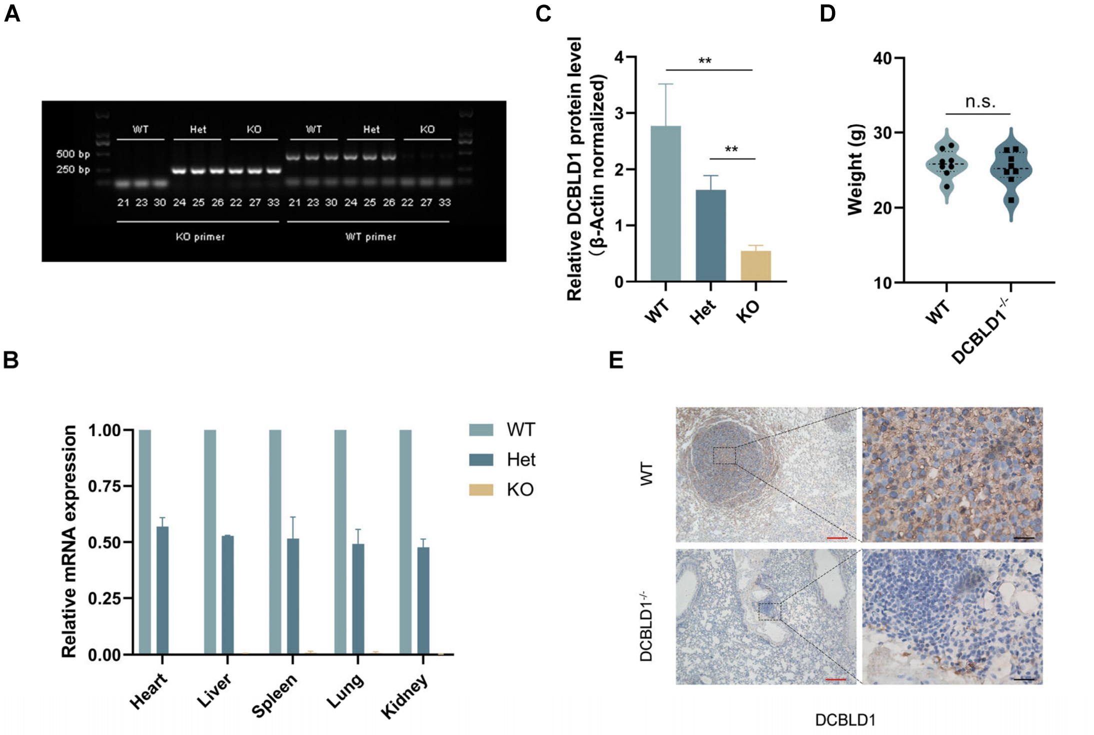

Supplementary Figure S3. Characterization and validation of DCBLD1 knockout mouse model. **A**. PCR-based genotyping analysis confirming the successful generation of DCBLD1 knockout (KO) mice. **B**. Quantitative real-time PCR analysis of DCBLD1 mRNA level in DCBLD1 wild-type (WT), heterozygous (Het), and KO mice. **C.** Quantitative analysis of DCBLD1 protein levels normalized to β-Actin in DCBLD1 WT, Het, and KO mice. **D**. Body weight comparison between DCBLD1 WT and KO mice following 30 weeks of urethane treatment. **E.** IHC staining of DCBLD1 in lung sections from DCBLD1 WT and KO mice. Red scale bars, 400 μm, Black scale bars, 40 μm. n.s., not significant; $^{**}p < 0.01$.

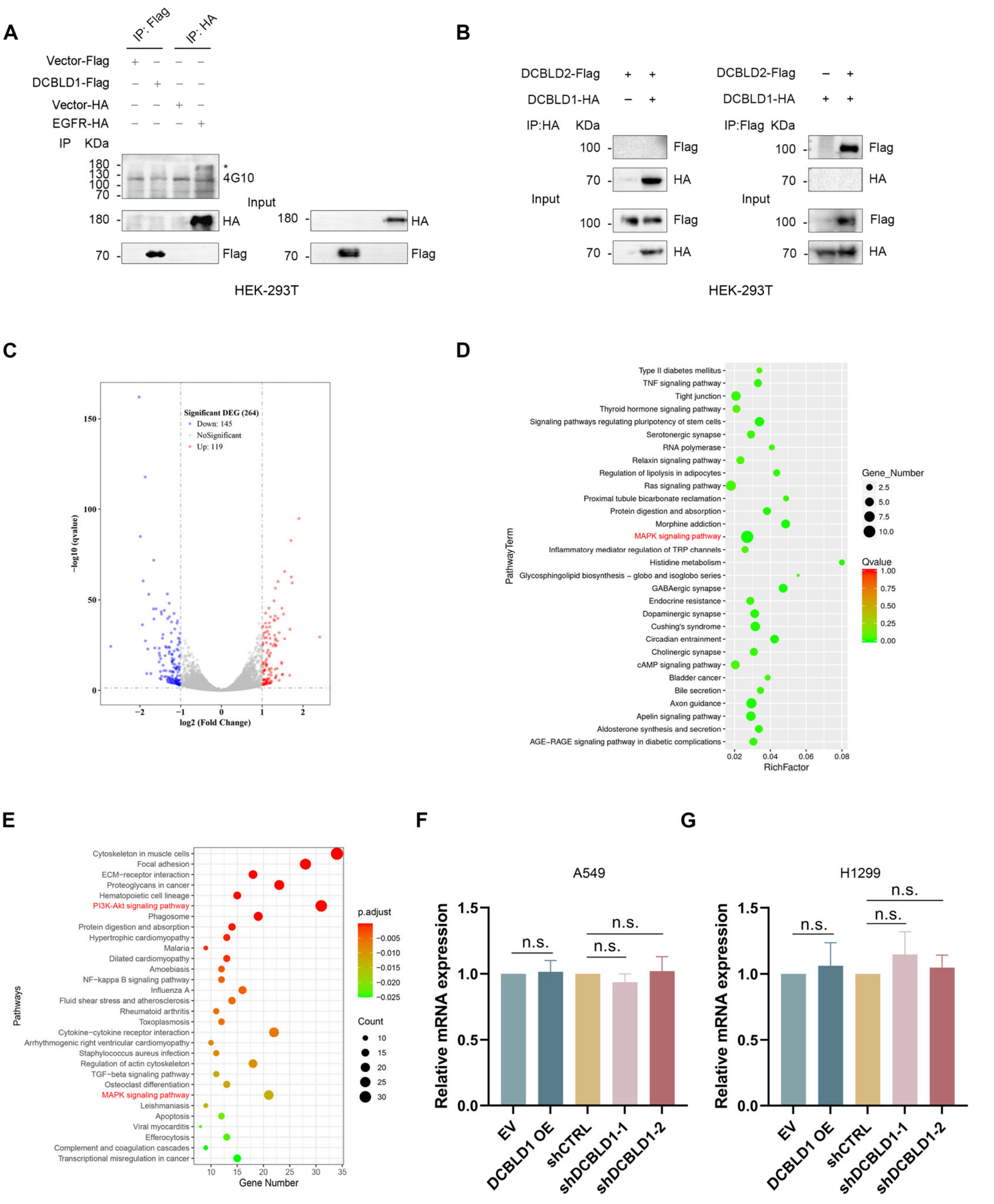

Supplementary Figure S4. DCBLD1 regulates EGFR signaling independently of EGFR transcription in LUAD cells. **A.** HEK293T cells were transfected with DCBLD1-Flag, EGFR-HA or vector plasmids as indicated and cultured for 48 h. Immunoprecipitation was performed using Flag or HA antibodies, followed by Western blot analysis with 4G10 anti-phosphotyrosine antibody to detect tyrosine phosphorylation levels. HA and Flag antibodies were used to confirm successful immunoprecipitation of target proteins. * indicate phosphorylated EGFR bands. **B.** HEK-293T cells were transfected with DCBLD2-Flag, DCBLD1-HA, or co-transfected with both constructs for 48 h. Co-immunoprecipitation assays were performed using Flag or HA antibodies. Immunoprecipitated protein complexes were analyzed by Western blot with Flag and HA antibodies to assess protein-protein interactions. **C.**

Volcano plot depicting differentially expressed genes (DEGs) identified by RNA-sequencing analysis comparing A549 cells expressing control shRNA (shCTRL) versus DCBLD1-targeting shRNA (shDCBLD1). **D.** KEGG pathway enrichment analysis of identified DEGs. **E.** KEGG pathway enrichment analysis of DCBLD1-correlated genes in the TCGA-LUAD dataset. **F**-**G.** Quantitative analysis of EGFR mRNA expression in A549 (**F**) and H1299 (**G**) cells following DCBLD1 overexpression or knockdown. n.s., not significant.

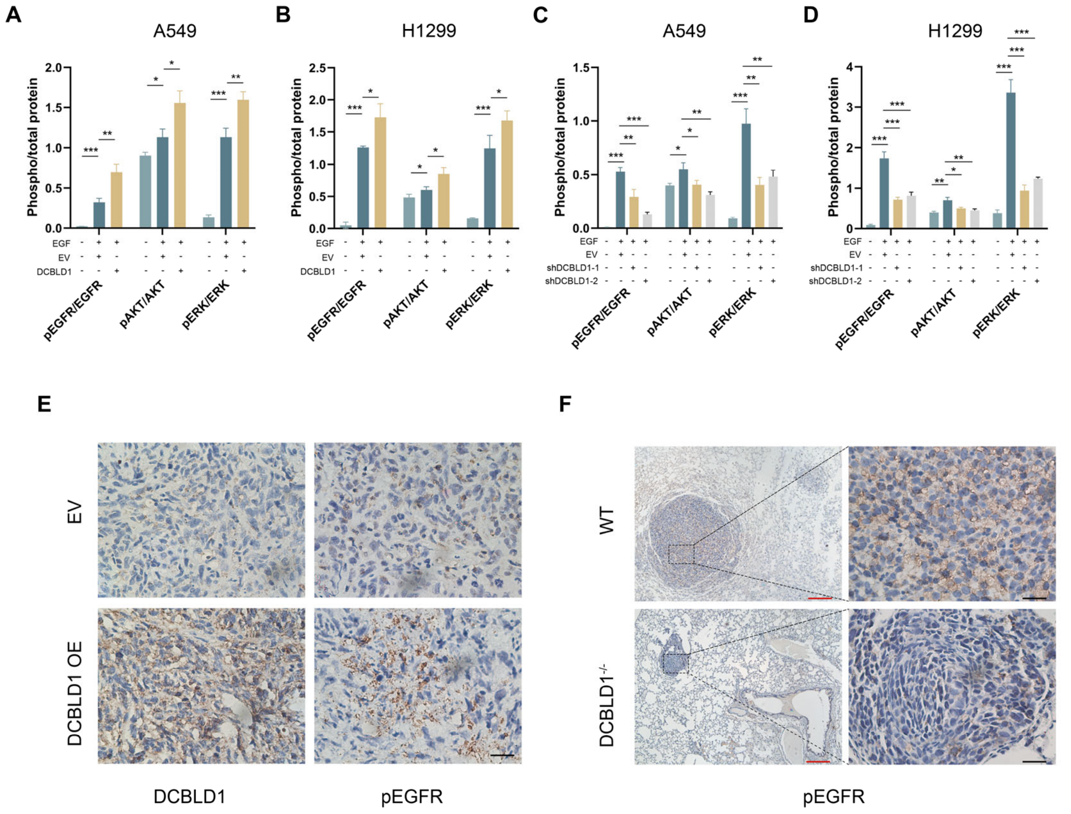

Supplementary Figure S5. DCBLD1 modulates EGFR phosphorylation and downstream signaling cascade in vitro and in vivo. **A**-**B.** Quantification of phospho to total protein ratios (pEGFR/EGFR, pAKT/AKT, pERK/ERK) in A549 (**A**) and H1299 (**B**) cells upon DCBLD1 overexpression. **C**-**D.** Quantification of phospho to total protein ratios (pEGFR/EGFR, pAKT/AKT, pERK/ERK) in A549 (**C**) and H1299 (**D**) cells following DCBLD1 knockdown. **E**. Representative IHC staining of DCBLD1and pEGFR in xenograft tumor sections from mice bearing DCBLD1-overexpressing and EV control tumors. Scale bar, 40 μm. **F.** Representative IHC staining of pEGFR in lung tissue sections from DCBLD1 wide-type (WT) and DCBLD1$^{-/-}$ mice. Red scale bars: 400 μm, Black scale bars:40 μm. $^{*}p < 0.05$, $^{**}p < 0.01$, $^{***}p < 0.001$.

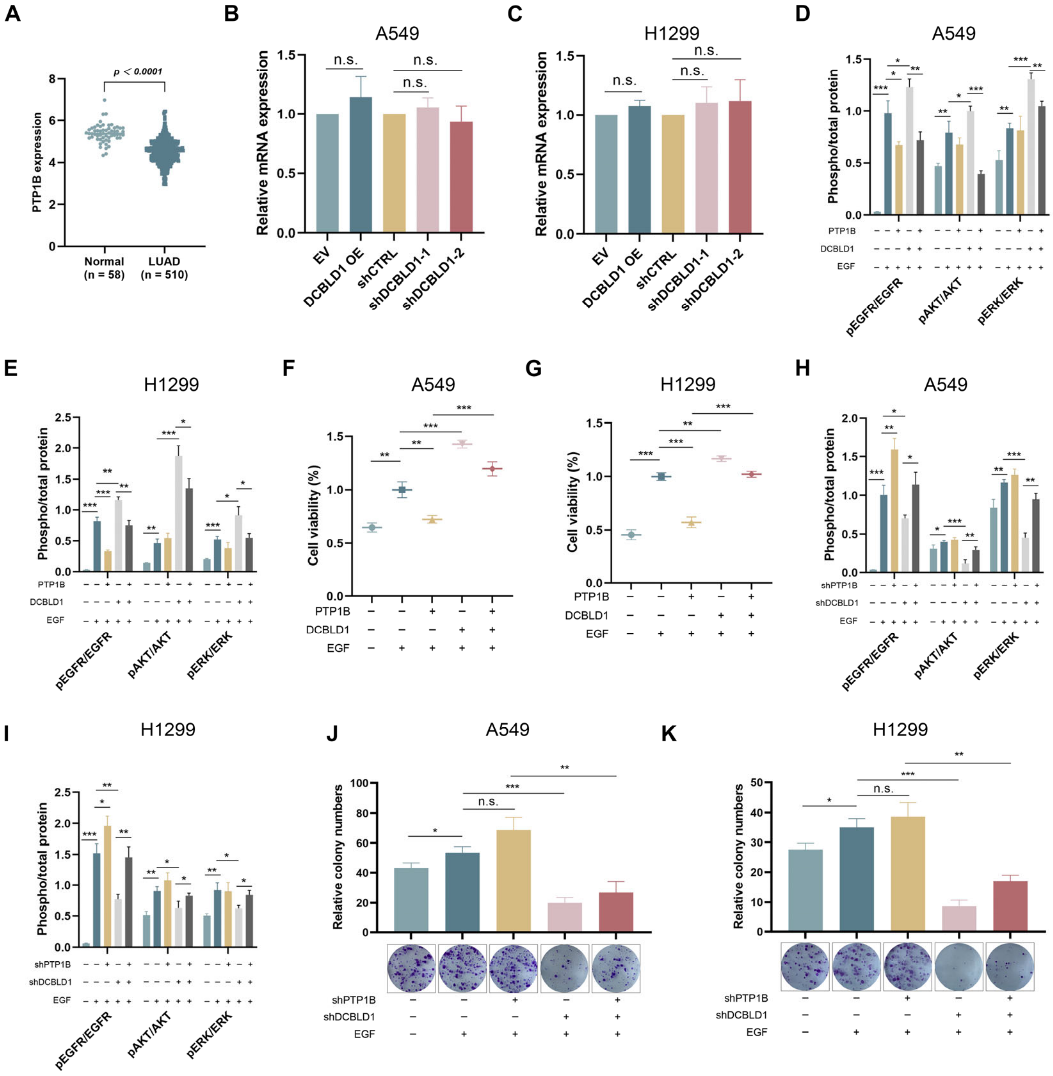

Supplementary Figure S6. DCBLD1 promotes LUAD progression by counteracting PTP1B. **A**. Analysis of PTP1B mRNA expression in LUAD tissues compared to adjacent normal lung tissues from TCGA datasets. **B-C**. Quantification of PTP1B mRNA levels in A549 (**B**) and H1299 (**C**) cells following DCBLD1 overexpression or knockdown. **D-E.** Quantification of phospho to total protein ratios (pEGFR/EGFR, pAKT/AKT, pERK/ERK) in A549 (**D**) and H1299 (**E**) cells expressing DCBLD1, PTP1B, both or neither. **F-G.** Cell viability measured by CCK-8 assay in A549 (**F**) and H1299 (**G**) cells expressing DCBLD1, PTP1B, both or neither. **H-I.** Quantification of phospho to total protein ratios (pEGFR/EGFR, pAKT/AKT, pERK/ERK) in A549 (**H**) and H1299 (**I**) cells expressing shDCBLD1, shPTP1B, both or neither. **J-K.** Colony formation assay in A549 (**J**) and H1299 (**K**) cells expressing shDCBLD1, shPTP1B. n.s., not significant; $^{*}p < 0.05$, $^{**}p < 0.01$, $^{***}p < 0.001$.

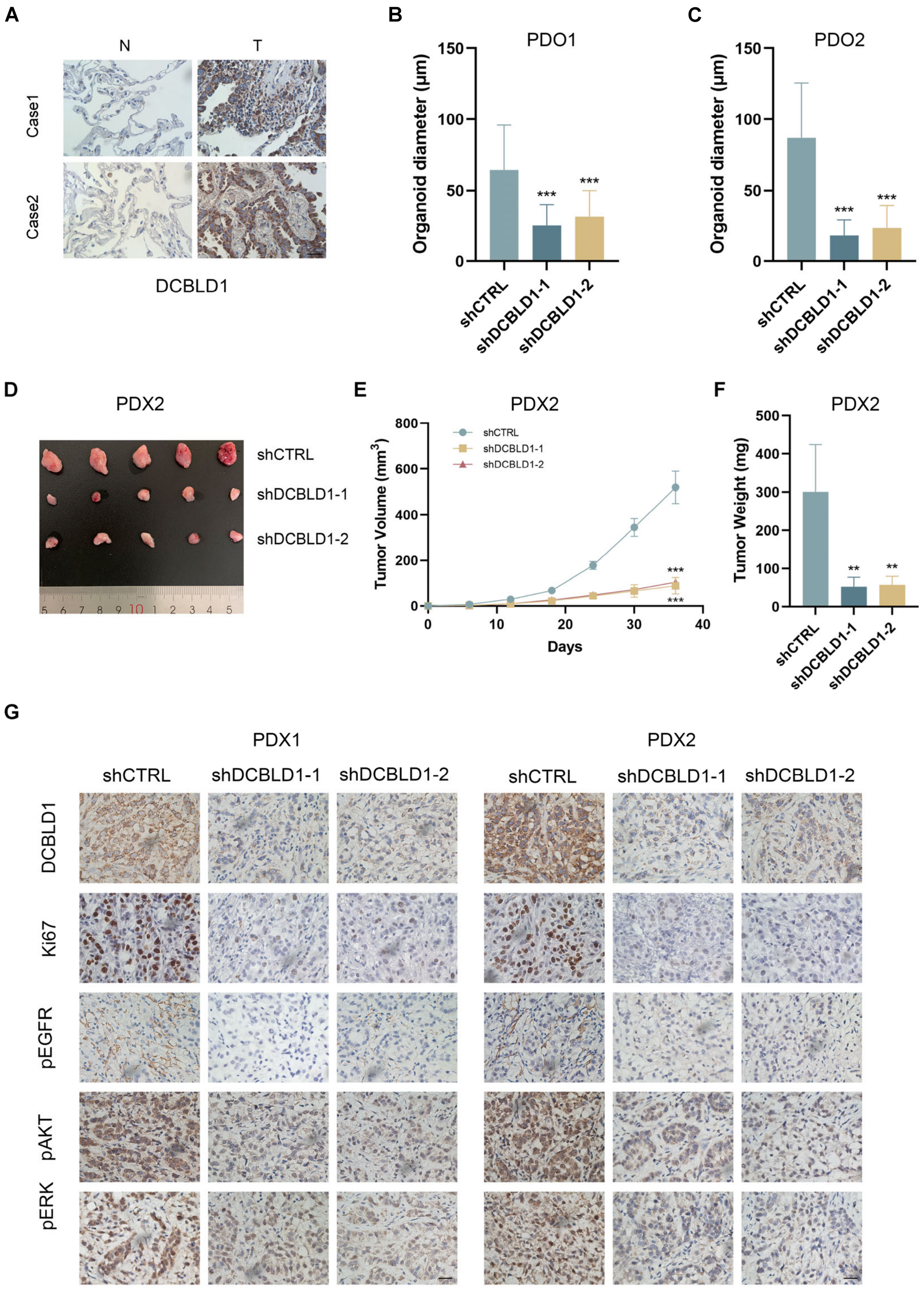

Supplementary Figure S7. DCBLD1 knockdown inhibits tumor growth in LUAD PDOs and PDXs, regardless of EGFR mutation status. **A**. Representative IHC staining of DCBLD1 in paired tumor and adjacent normal tissues from EGFR wild type (WT, Case1) and EGFR mutant (Mut, Case2) LUAD specimens. Scale bar, 40 μm. **B**-**C**. Quantification of organoid size from PDO1 (**B**) and PDO2 (**C**) treated with shCTRL or shDCBLD1. **D**-**F**. Gross morphological images (**D**), relative tumor volume (**E**), and tumor weight (**F**) of PDX2 treated with shCTRL or

shDCBLD1. Tumor volume was determined by caliper measurements using the formula (length × width^2)/2. **G**. Representative IHC images of DCBLD1, Ki67, pEGFR, pAKT and pERK in PDX1 and PDX2 tumor sections. Scale bar, 40 μm. $^{**}p < 0.01$, $^{***}p < 0.001$.

Supplementary Table S1. Primers used in this study

Gene	Primer sequence	Application
DCBLD1	F:GCTTTCTGCTGACCTACGCCAG<br>R:ATCTCCTGCTATGTCTCTACAGCCAG	Mouse qPCR primer pair
DCBLD1	F:ACACAAGTGAAGTAACCGTCCG<br>R:TGCTACGTCTCTACAACCAGC	Human qPCR primer pair
Actin	F:GGCTGTATTCCCCTCCATCG<br>R:CCAGTTGGTAACAATGCCATGT	Mouse qPCR primer pair
Actin	F:GACAGGATGCAGAAGGAGAT<br>R:GAGGCCAGGATGGAGC	Human qPCR primer pair
DCBLD1<br>-KO	F:ACCACATGGGCACCATTCCTAA<br>R:TTCCCTTTCTCAGTGGTCTTCC	Mouse PCR primer pair for genotyping
DCBLD1<br>-WT	F:CCTATTGCCCTGACTCATGCAA<br>R:TACTGAGCATCTTGCCTCCAGT	Mouse PCR primer pair for genotyping
EGFR<br>-5'arm	F:GGGCAGTCTGGTACTTCCAAGCT<br>R:TGGCGTTACTATGGGAACATACGTC	Mouse PCR primer pair for genotyping
EGFR<br>-WT	F:CAGCAAAACCTGGCTGTGGATC<br>R:ATGAGCCACCATGTGGGTGTC	Mouse PCR primer pair for genotyping
Cre<br>-3'arm	F:GGCTGGACCAATGTGAACATTG<br>R:TAACACCCGTGTATGGCACCC	Mouse PCR primer pair for genotyping
Cre<br>-WT	F:TGCTTCACAGGGTCGGTAGAAAC<br>R:TAACACCCGTGTATGGCACCC	Mouse PCR primer pair for genotyping
EGFR	F:AGGCACGAGTAACAAGCTCAC<br>R:ATGAGGACATAACCAGCCACC	Human qPCR primer pair
PTP1B	F:TGTCTGGCTGATACCTGCCTCT<br>R:ATCAGCCCCATCCGAAACTTCC	Human qPCR primer pair
DCBLD1	F:TCCATAGAAGATTCTAGAGCCACCATGGTGCCC GGCGCCCGC<br>R:CGATTTAAATTCGAATTCTTATTTGTCGTCATCA TC	Human PCR primer pair for Flag tagged DCBLD1 cloning
DCBLD1<br>-ECD	F:GATCTTTGCAGCCTTTGACTACAAGGATGACGAT GACAAG<br>R:GTCATCCTTGTAGTCAAAGGCTGCAAAGATCCC CAT	Human PCR primer pair for Flag tagged DCBLD1 ECD cloning
DCBLD1<br>-ICD	F:TCTAGAGCCACCATGAGAAAGAAGAAGAAGAA AGGAAGTC<br>R:CTTCTTCTTCTTTCTCATGGTGGCTCTAGAATCTT	Human PCR primer pair for Flag tagged DCBLD1 ICD cloning
shDCBLD<br>1-1	F:CCGGCGGAAGAAACATCCACAGGAACTCGAGTT CCTGTGGATGTTTCTTCCGTTTTTG<br>R:AATTCAAAAACGGAAGAAACATCCACAGGAACT CGAGTTCCTGTGGATGTTTCTTCCG	Human shRNA primer pair for DCBLD1 knockdown

shDCBLD 1-2	F:CCGGCAGCGACCATCCAGATTTAATCTCGAGATTAAATCTGGATGGTCGCTGTTTTTG<br>R:AATTCAAAAACAGCGACCATCCAGATTTAATCTCGAGATTAAATCTGGATGGTCGCTG	Human shRNA primer pair for DCBLD1 knockdown
EGFR	F:AGCCCGGGCGGATCCAAGCTTATGCGACCCTCCGGGAC<br>R:GTATGGGTATCTAGACTCGAGTGCTCCAATAAATTCACTGCTTTG	Human PCR primer pair for HA tagged EGFR cloning
EGFR -ECD	F:ATCGGCCTCTTCATGTACCCATACGATGTTCCAGATT<br>R:ATCGGCCTCTTCATGTACCCATACGATGTTCCAGATT	Human PCR primer pair for HA tagged EGFR ECD cloning
EGFR -ICD	F:GGATCCAAGCTTATGCGAAGGCGCCACATCGTT<br>R:GATGTGGCGCCTTCGCATAAGCTTGGATCCGCC	Human PCR primer pair for HA tagged EGFR ICD cloning
PTP1B	F:GGCTAGCGTTCTCGAGAATTCATGGAGATGGAAAAGGAGTTCG<br>R:GATCCAAGCGGCCGCTCTAGACTACTTGTCATCGTCGTCCTTGTAATCTGTGTTGCTGTTGAACAGG	Human PCR primer pair for Flag tagged PTP1B cloning
shPTP1B	F:CCGGTGCGACAGCTAGAATTGGAAACTCGAGTTTCCAATTCTAGCTGTCGCATTTTTG<br>R:AATTCAAAAATGCGACAGCTAGAATTGGAAACTCGAGTTTCCAATTCTAGCTGTCGCA	Human shRNA primer pair for PTP1B knockdown

Supplementary Table S2. Antibodies used in this study

Antigen	Company	Cat. No.	Dilution for WB	Dilution for IHC/IF
DCBLD1	Proteintech	24504-1-AP	1:2000	1:200
EGFR	Abcam	ab289889	1:5000	1:400
p-EGFR	Abcam	ab40815	1:2000	1:200
ERK	Cell Signaling Technology	4695	1:2000	
p-ERK	Cell Signaling Technology	4370	1:2000	1:200
AKT	Abmart	T55561	1:2000	
p-AKT	Abmart	T40067	1:2000	1:200
PTP1B	Abmart	T56593	1:2000	
TTF-1	Abcam	ab76013		1:200
NAPSIN	Cell Signaling Technology	62434		1:200
Ki67	Cell Signaling Technology	9449		1:500
Flag	Abmart	M20008	1:5000	
HA	Abcam	ab9110	1:5000	
4G10	Sigma Aldrich	05-321	1:1000	
β-Actin	YEASEN	30101ES	1:5000	
Rabbit IgG (H+L), Cy3-labeled	YEASEN	33108ES		1:500
Rabbit IgG (H+L)	Proteintech	SA00001-2	1:10000	1:1000
Mouse IgG (H+L), FITC-labeled	YEASEN	33207ES		1:500
Mouse IgG (H+L)	Proteintech	SA00001-1	1:10000	1:1000
CD31 APC	BD Pharmingen	551262		
CD45 RB705	BD Pharmingen	570291		
CD326 (Ep-CAM) PE	BD Pharmingen	563477		
